# Modeling grain biochemical composition traits of commercial sorghum hybrids under diverse management practices

**DOI:** 10.3389/fpls.2026.1768456

**Published:** 2026-02-16

**Authors:** Boubacar Gano, Marie de Gracia Coquerel, Jocelyn Saxton, Nathaniel Eck, Kamaranga H. S. Peiris, Scott R. Bean, Jaccob Stanton, Nurzaman Ahmed, Nadia Shakoor

**Affiliations:** 1 Donald Danforth Plant Science Center, Saint Louis, MO, United States; 2 Grain Quality and Structure Research Unit, Center for Grain and Animal Health Research, USDA-ARS, Manhattan, KS, United States

**Keywords:** agricultural practices, grain composition, grain quality, machine learning, regression models, *Sorghum bicolor*, sustainable agriculture

## Abstract

**Introduction:**

Sorghum (*Sorghum bicolor* (L.) Moench) is a vital cereal crop for food, feed, and biofuel production. Accurate estimation of grain biochemical composition, crude protein (CP), lysine from grain (LysG) and protein (LysP), starch (SC), amylose from grain (AMLG) and starch (AMLS), and crude fat (CF), is crucial for improving breeding and management strategies. Our aim is not pre-harvest forecasting but reducing laboratory cost by identifying a minimal set of post-harvest measurements required to estimate other grain composition traits accurately.

**Methods:**

We used machine learning (ML) models to predict grain quality traits in commercial sorghum hybrids under different management practices, including precision nitrogen application, cover cropping, and no-till methods. Multi-year field trials (2023–2024) in Saint Charles, Missouri, integrated agronomic, physiological, UAV-based, and environmental data for model training and validation.

**Results:**

Phenotypic analysis showed that grain composition traits varied significantly by year and management practices. Among ML models, LASSO and ElasticNet achieved the highest predictive accuracy for crude protein (R² = 0.90) and amylose content (AMLS, R² = 0.99; AMLG, R² = 0.92). Bayesian Ridge was most effective for lysine from protein (R² = 0.64), while Partial Least Squares (PLS) excelled in starch content prediction (R² = 0.80). The correlation between grain composition (LysP, CF) and photosystem II efficiency (PhiPS2) indicated that enhanced photosynthesis and yield promote their accumulation. However, Partial Dependence Plots (PDPs) revealed strong non-linear effects, where slight variations in leaf temperature (Tleaf) and stomatal conductance (gsw) were associated with significant shifts in amylose content.

**Discussion:**

This study highlights the role of genotype × management interactions in sorghum breeding and demonstrates the value of integrating ML-driven models to enhance grain quality and precision agriculture strategies.

## 1 Introduction

Sorghum (*Sorghum bicolor* L. Moench) is a versatile cereal crop widely cultivated across diverse agro-climatic regions due to its adaptability to harsh environmental conditions, including drought and high temperatures (Tuinstra et al., 1997). Beyond its traditional use as a staple food in many parts of Africa and Asia and as a feed grain in many parts of the world, sorghum is gaining attention for its industrial applications, particularly in grain ethanol production and brewing (Murray et al., 2008; Boyles et al., 2016; Ahmad Dar et al., 2018). The growing demand for sorghum intensifies the need for enhanced hybrids with superior grain biochemical composition, such as high protein and starch content, which are key determinants of its nutritional and economic value (Sukumaran et al., 2012; Tanwar et al., 2023). Modeling these biochemical traits under different management practices offers a promising approach to accelerate the breeding and selection process, enabling the development of commercially viable hybrids with desirable characteristics (Hammer et al., 2019).

The biochemical composition of sorghum grain is shaped by both genetic and environmental factors, with management practices such as fertilization, tillage, and cover cropping playing key roles in determining yield and grain quality (Reddy et al., 2003). Traditional assessments of grain composition traits (e.g. protein, starch, etc.) using laboratory-based spectroscopy are often labor-intensive, time-consuming, and costly, limiting the speed of hybrid development (Peiris et al., 2020, 2021). Advances in high-throughput phenotyping, coupled with machine learning and statistical modeling, now enable accurate estimation of grain composition traits from drone imagery, environmental variables, and management data. The models can integrate diverse datasets, including soil properties, weather patterns, and agronomic inputs, to capture interactions that shape sorghum grain composition. Machine-learning algorithms such as LASSO, ElasticNet (ELN), Partial Least Squares (PLS), Random Forest (RF), and Extreme Gradient Boosting (XGBoost) have proven effective in modeling because they capture both linear and non-linear relationships in complex data (Adak et al., 2024). When trained on past seasons’ data, these models can predict grain composition with high accuracy, supporting sorghum breeding and cultivation strategies (Shakoor et al., 2017).

Field experiments with commercial sorghum hybrids under diverse management practices provided the primary dataset for developing models. These trials also enabled robust evaluation of the relationships between management and grain biochemical traits. Such insights can assist breeders and farmers in identifying optimal management strategies that maximize the nutritional quality and yield of sorghum hybrids. Measurements include grain biochemical traits, such as crude protein (CP), starch (SC), amylose (AML), Lysine (Lys), and crude fat (CF), alongside management factors like precision nitrogen, cover crop, and no-till practices. The models were trained using data on agronomic traits such as yield, weather conditions, and field management practices, including nitrogen and herbicide applications.

Several research studies have demonstrated the efficacy of machine learning algorithms in predicting grain composition traits across different crops. In maize (*Zea mays* L.), for example, studies by (Cataltas and Tutuncu, 2023; Ahmed and Kamruzzaman, 2024; Rodrigues et al., 2024) have utilized ML models to predict grain quality, including protein, oil, and starch content, using near infrared spectroscopy and environmental sensor data. Random Forest (RF), Partial Least Squares (PLS), and Convolutional Neural Network (CNN) have shown high predictive accuracy in these studies, underscoring the potential of these models in complex trait prediction.

In wheat (*Triticum aestivum* L.), predictive modeling has been employed to estimate grain protein and gluten content based on hyperspectral imaging data (Ma et al., 2022). Research conducted by (Caporaso et al., 2018; Zhang et al., 2024) highlighted the effectiveness of RF and PLS models, in extracting features from spectral data to enhance prediction accuracy. Similarly, soybean (*Glycine max* L.) research by Yakubu et al. (2024) has utilized the PLS model for predicting oil and protein content from near-infrared (NIR) spectroscopy data, demonstrating the versatility of machine learning approaches in handling diverse data modalities.

Specific to sorghum, recent studies by Nazari et al. (2022) have applied ML models to predict starch, protein, and phenolic compound concentrations using image analysis and machine learning. In addition, genomic prediction models, such as Genomic Best Linear Unbiased Prediction and Bayesian Multi-Output Regression Stacking (BMORS), have been combined with environmental covariates to improve prediction accuracy of sorghum grain composition (Sapkota et al., 2020). Furthermore, the integration of ML models with hyperspectral data has facilitated the non-destructive estimation of grain nutrient content in sorghum, enabling real-time monitoring and decision-making in precision agriculture (Wu et al., 2025).

The advancement of high-throughput phenotyping platforms, including drone-based multispectral imaging and ground-based spectroscopy, has further augmented the potential of predictive modeling in sorghum breeding. Studies by many authors (Caporaso et al., 2018; Ma et al., 2022; Zhang et al., 2024) have shown that these platforms generate large volumes of data, which, when coupled with robust ML algorithms, can lead to significant improvements in the accuracy and scalability of biochemical trait estimation. Additionally, the incorporation of feature selection techniques, such as Recursive Feature Elimination (RFE) and LASSO regression, has been instrumental in identifying key predictors and reducing model complexity.

Previous research in sorghum has successfully applied near-infrared spectroscopy (NIRS), indoor hyperspectral imaging, and related laboratory-based methods to estimate grain quality traits (Bean et al., 2006; Sapkota et al., 2020; Peiris et al., 2021; Nazari et al., 2022). However, most of these prior works have relied on well-established laboratory-based measurements, which, while powerful, can be costly, require specialized equipment, and become impractical when evaluating large breeding populations or a wide range of biochemical traits. The novel contribution of our study is the integration of in-field physiological, morphological, and agronomic measurements collected throughout the growing season, together with UAV-derived indices to estimate key grain biochemical traits such as crude protein, lysine, starch, amylose, and crude fat. By linking these agro-physiological datasets to key grain biochemical traits across multiple management regimes (precision nitrogen application, cover cropping, tillage, and their combinations), we aim to demonstrate a scalable and cost-effective framework for grain quality modeling.

Unlike pre-harvest forecasting studies, we use all traits, including other grain composition traits as predictors for a given target. Our objective is to minimize the number of laboratory assays: if routinely measured protein content enables accurate estimation of amylose, starch, lysine, and related traits, those additional assays can be omitted. This approach advances sorghum machine learning research by shifting reliance away from extensive laboratory reference datasets toward models that can be calibrated with minimal reference measurements, thereby improving both efficiency and accessibility for breeding and production systems.

## 2 Materials and methods

### 2.1 Field trials design and conditions

**Figure 1:**
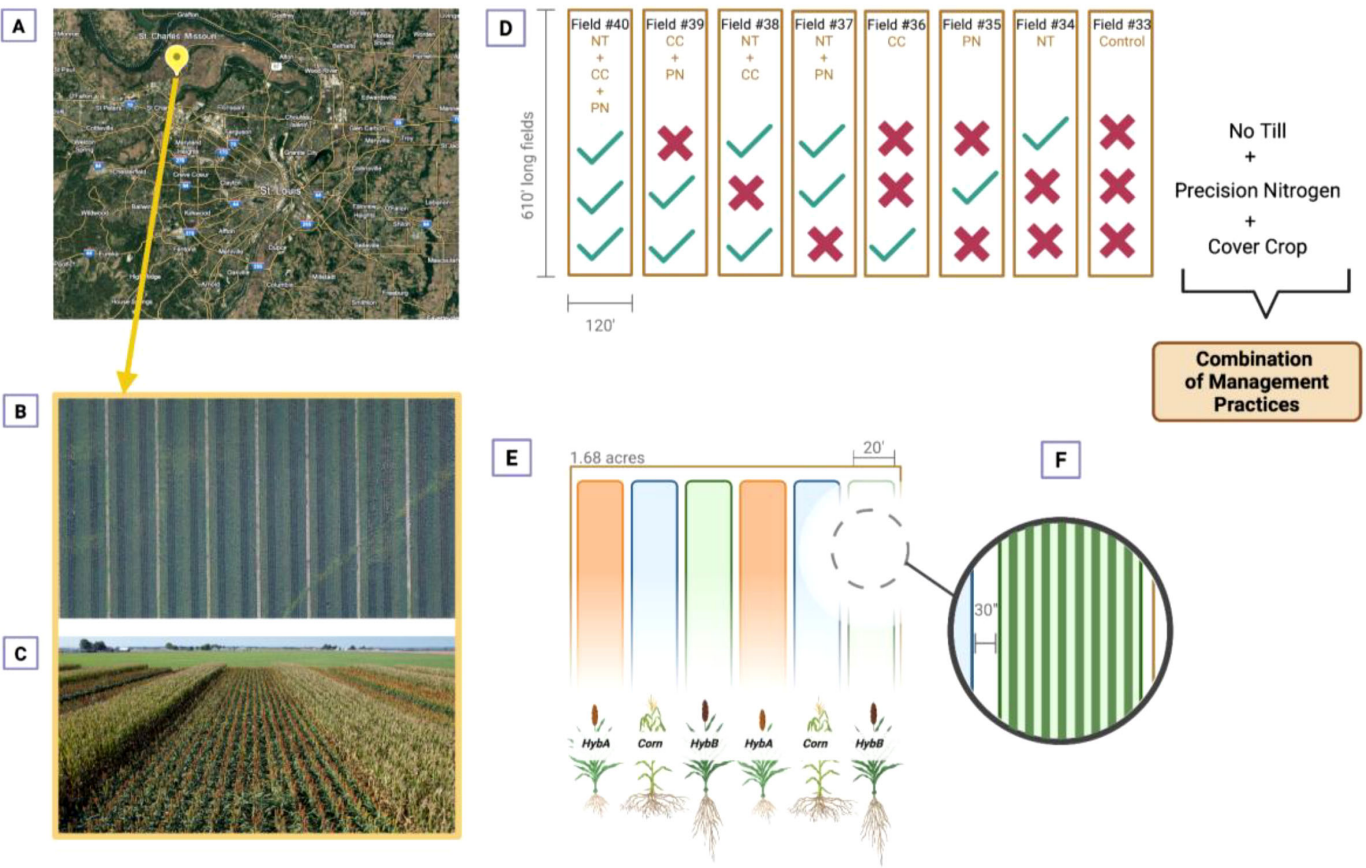
Field layout and experimental design for evaluating sorghum and corn responses to integrated agronomic practices. **(A)** Geographic location of the experimental site is near Saint Charles, Missouri. **(B)** Aerial view of the field showing long rows used for the experiment. **(C)** Lateral view of the experimental plots. **(D)** Treatment combinations across fields, including No-Till (NT), Precision Nitrogen (PN), and Cover Cropping (CC), with specific fields following different combinations of these practices. **(E)** Plot layout within the experimental area, showing alternating sections planted with different sorghum hybrids (HybA, HybB) and corn. **(F)** Detailed zoom-in of individual rows within the plot.

Field experiments were conducted in 2023 and 2024 at the Danforth Field Research Site (FRS) in Saint Charles, Missouri (38.8471906, -90.4493288), where we are implementing a five-year field trial that includes two commercial sorghum varieties (Hybrid A and Hybrid B) intercropped with a regional corn variety across 5.4 ha (**Figures 1A–C**). The site was divided into eight subfields, each assigned a combination of management practices: no-till, precision nitrogen, and winter cover cropping (**Figure 1D**). A split-plot design was implemented where sorghum hybrids were grown on main plots measuring 6 m × 186 m within fields of 36 m × 186 m (0.67 ha) (**Figures 1D–F**). Each management × hybrid combination was represented by two replicate plots per year, randomized within subfields to minimize positional bias. The fields are located on a consistent DeSioux loam soil type in a flood-free zone (USDA Web Soil Survey), with a mean annual precipitation of 1,074 mm (St. Charles climate: Weather St. Charles & temperature by month, n.d.). We recognize that confounding between management and subfield location may limit inference about pure management effects; however, replication across space within subfields and across time strengthens our ability to detect consistent hybrid and management responses. Management and subfield are not independently randomized, and therefore management effects should be interpreted as conditional on subfield context rather than as isolated causal effects. This limitation was addressed analytically by emphasizing multi-year consistency and validation-based model robustness rather than formal fixed- or random-effect inference for management.

Planting occurred in June 2023 for the first experiment and in May 2024 for the second. Harvest followed in November 2023 and October 2024, respectively. Herbicide treatments were applied in both years for weed control (**Supplementary Table S1**). Irrigation was applied only during persistent drought periods, which occurred in mid-July in 2023 and late June 2024. Fields were harvested at ≤15% grain moisture. Post-harvest, staff mowed the fields to reduce stubble and facilitate plant decomposition before the next growing season.

**Figure 2:**
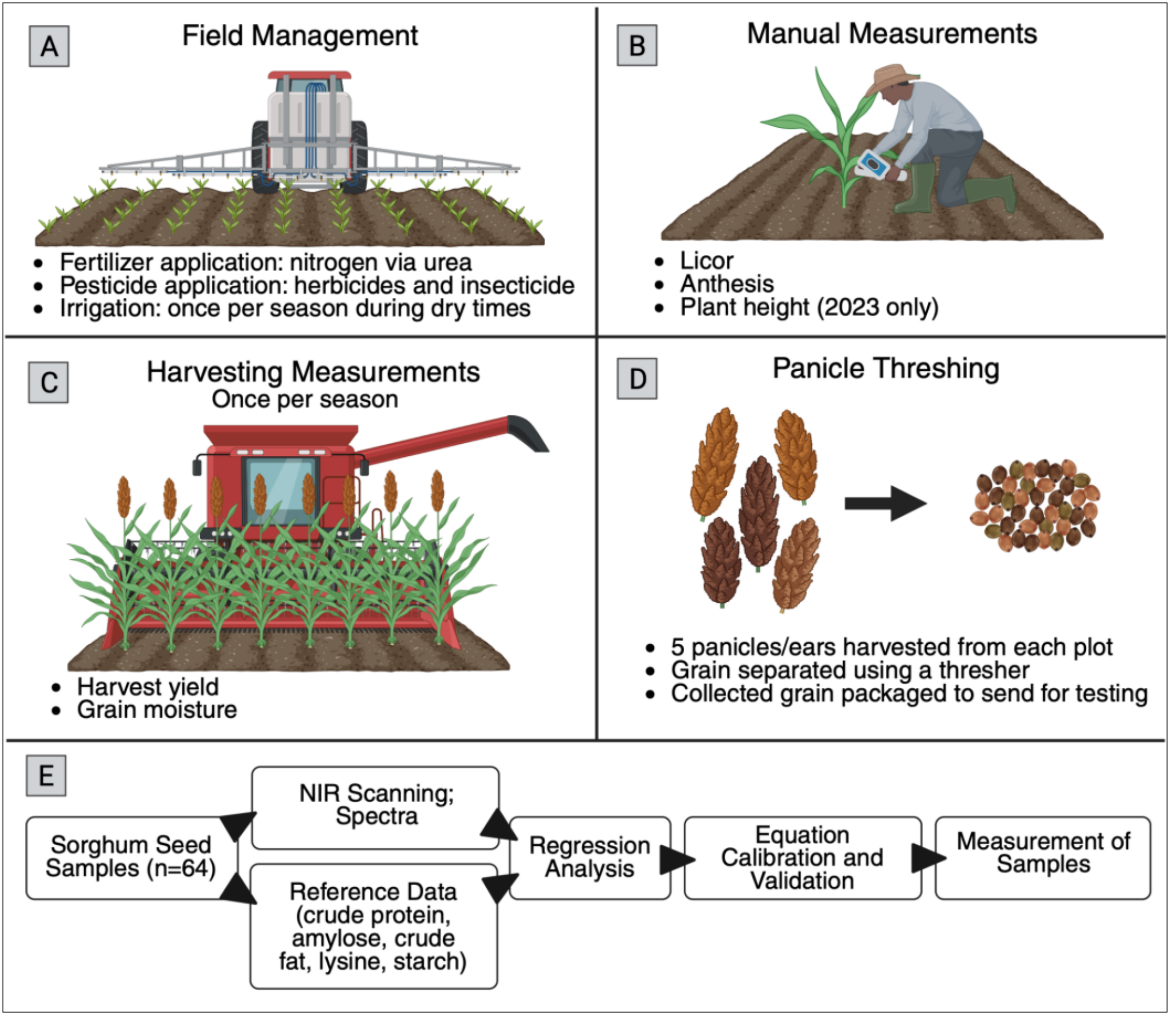
Overview of sorghum field management, phenotyping, and grain quality assessment pipeline. **(A)** Field management depicting fertilization, herbicide application, and irrigation practices; **(B)** Manual measurements showing vegetative stage assessment and plant height measurements; **(C)** Harvesting measurements displaying yield and moisture data collection; **(D)** Panicle threshing demonstrating grain separation process; and **(E)** near-infrared (NIR) spectroscopic analysis workflow for determining grain composition including crude protein, amylose, crude fat, lysine, and starch content. Created in BioRender (Shakoor, 2025).

Management practices were a key component of the experiment. Tillage, where applied, was performed within one month before planting. Conventional nitrogen was applied at a total rate of 224 kg ha^-1^ in two 112 kg ha^-1^ splits: one before planting and one about a month afterward, following Sorghum Checkoff recommendations (Nitrogen, Fertilizer Choices and Application, 2017). No-till (NT) practices were used in designated fields, involving crop establishment without soil disturbance to preserve soil structure, moisture, and organic matter. In precision nitrogen (PN) fields, soil samples were collected 5–6 weeks before planting and analyzed at the University of Missouri Extension Soil and Plant Testing Laboratory. Nitrogen application rates were calculated as: PN rate = (9,415.15 kg ha^-1^ yield goal × 1.6) − (soil organic matter % × 20) − soil N (kg ha^-1^) (Available online at: https://www.sorghumcheckoff.com/wp-content/uploads/2021/11/2018_04_17_CentralEasternPlainsGuide.pdf). Precision rates were lower than conventional in all cases and were applied in two splits at the same timings as conventional N. Hairy vetch (*Vicia villosa*) was used as the cover crop (CC) for its nitrogen-fixing properties and was planted in mid-October in designated fields. Control plots (C) received conventional tillage and conventional nitrogen application, serving as a baseline for comparison with conservation practices (**Figure 2A**).

### 2.2 Data collection

#### 2.2.1 Weather data collection

To monitor environmental conditions during the trials, we integrated two complementary real-time data sources: the NASA POWER API (NASA POWER Homepage, n.d.) and the PheNode environmental sensor platform (Agrela Ecosystems) (PheNode, n.d.). The NASA POWER API provided weather data, including temperature, relative humidity, solar radiation, and precipitation, at specific latitude–longitude coordinates within defined date ranges (June 1 to October 30). We configured the API to return hourly means for our Saint Charles, MO field site and seasonal averages for additional sorghum-growing states and implemented a retry mechanism to handle network or server interruptions. These data were parsed from JSON into Pandas DataFrames for downstream analysis.

The PheNode system was deployed mid-season in 2023 at the Danforth Field Research Site, where it continuously recorded on-site atmospheric conditions (air temperature, humidity, solar radiation, and precipitation) from June through October, covering critical sorghum growth stages. Because PheNode was installed after the 2023 field trial had already begun, and because occasional data gaps arose from either source, the two systems served as complements: PheNode captured detailed, site-specific conditions, while the NASA API both filled missing data windows and extended coverage to additional sorghum production regions across the United States.

#### 2.2.2 Physiological traits assessment (LI-600)

Physiological trait data were collected from sorghum plants at vegetative stage using the LI-600 Porometer-Fluorometer (Licor, Lincoln, NE, USA), a handheld device that measures photosynthetic and water-use efficiency in real-time (**Figure 2B**). Sampling was systematically conducted across experimental plots to capture variation among genotypes, focusing on the youngest fully expanded leaves at consistent canopy positions to minimize variability. Key measurements included PhiPS2 (photosynthetic efficiency), Tleaf (leaf temperature), Qamb (ambient light intensity), VPDleaf (vapor pressure deficit at the leaf level), and leaf angle. Data was collected during the day between 9 AM and 1 PM to avoid heat stress artifacts. The LI-600 was calibrated to ensure accuracy, and all data was logged directly into the device for streamlined transfer to a central database. This approach enabled non-invasive phenotyping, providing critical insights into sorghum’s photosynthetic performance and water-use dynamics under field conditions.

#### 2.2.3 Harvest and agro-morphological traits assessment

For 2023, the first year of the trial, manual measurements of the plants occurred when the crops reached physiological maturity. In each plot, height, panicle exertion, and lodging were measured by hand. For these hybrid varieties, there was no lodging observed, and the height and panicle exertion were very uniform. Therefore, we omitted these manual measurements in the second year.

Plants were harvested when grain moisture reached ≤ 15%, corresponding to 151 days after planting in 2023 and 146 days in 2024. Grain moisture and yield were recorded during harvest in both seasons. In 2023, an external farmer conducted the harvest, and all eight rows of each plot were combined to obtain a single total grain weight, as the primary goal that year was commercial harvest rather than detailed yield partitioning. In contrast, the 2024 harvest was performed using a Danforth Center–owned Massey Ferguson Activa 7345 combine (Duluth, GA), which allowed for plot-level yield and test weight measurements (**Figures 2C, D**). That year, only the middle four rows of each plot were harvested, and the recorded yields were doubled to estimate total plot yield. Collecting yield data from the inner rows is a standard practice in agricultural field research to minimize edge effects; however, this approach was only feasible in 2024 when the lab had access to its own combine and complete control over the harvest process. Yield weights were converted from pounds to bushels per acre using the Grain Unit Converter tool (Combyne Ag) (Combyne, n.d.).

#### 2.2.4 Lab-based grain processing

Dry panicles were collected from each treatment, resulting in 64 samples (32 per year). Grain of both sorghum varieties were harvested at ≤ ;15% grain moisture. In 2023, 50-gram grain samples were analyzed, while in 2024, the sample size was increased to 100 grams to improve analytical precision and data reliability. All analyses were conducted at the Center for Grain and Animal Health Research (CGAHR), a USDA-ARS facility in Manhattan, KS.

NIR analyses were performed using a Perten DA7250 instrument, calibrated to parameters described by (Peiris et al., 2019, 2020, 2021). Each sample underwent three scans to predict crude protein (CP), lysine from protein (LysP) and grain (LysG), starch (SC), amylose from grain (AMLG), and starch (AMLS), and crude fat content (CF) (**Figure 2E**). Predictions were reported on a grain weight basis (g/100g grain); with lysine and amylose also calculated on a protein (g/100g protein) and starch (g/100g starch) basis, respectively (**Table 1**). To establish prediction accuracy, Mahalanobis distance (MD) was calculated for each sample and predicted trait. Physical grain traits for sorghum, such as mean kernel hardness index, mean kernel diameter, and mean kernel weight, were measured using the single kernel characterization system (Bean et al., 2006) with 100 kernels analyzed per replication.

**Table 1:** Calibration model statistics.

NIR	N	LV	R^2^	RMSECV	Slope	Min	Max
calibration							
Moisture	396	5	0.97	0.67	0.97	4.36	20.94
Protein	356	12	0.96	0.49	0.97	7.45	17.62
Lysine	1254	9	0.76	0.022	0.77	0.17	0.47
Starch	239	11	0.85	1.71	0.86	50.73	74.17
Amylose	499	11	0.82	2.62	0.83	0.25	27.9
CF	71	10	0.7	0.36	0.76	1.49	4.46

N, Number of calibration samples.

LV, Latent variables in the PLS model.

R^2^, Coefficient of determination of the PLS model.

RMSECV, Root mean square error of cross-validation.

Slope, Slope of the curve.

#### 2.2.5 UAV data collection and processing

**Figure 3:**
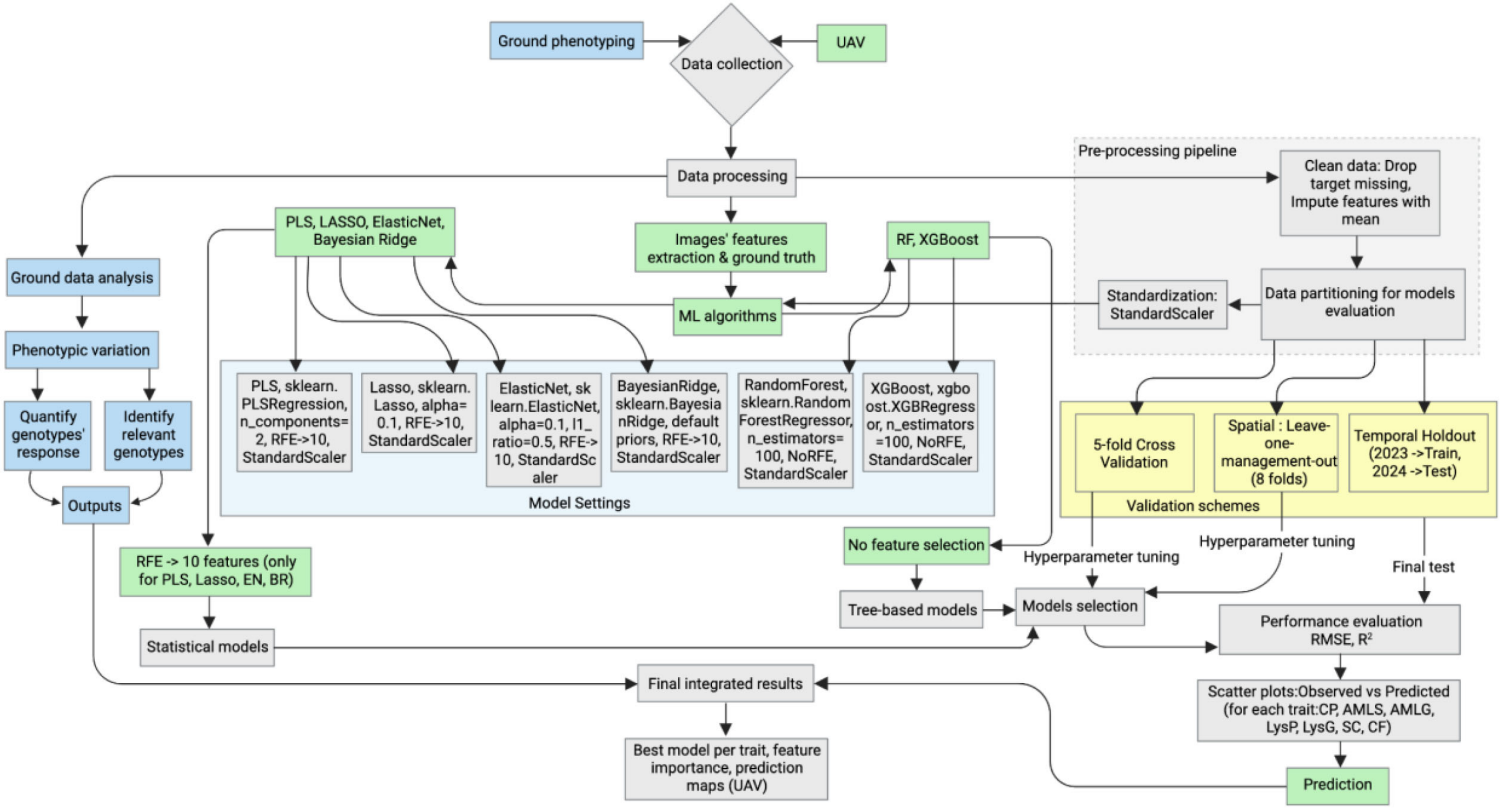
Integrated workflow for UAV data collection, processing, modeling and phenotypic analysis. This diagram illustrates the end-to-end analytical pipeline used in this study, beginning with ground phenotyping and UAV-based multispectral imaging, followed by data processing, feature extraction, preprocessing, and standardization. Multiple machine learning algorithms (PLS, LASSO, ElasticNet, Bayesian Ridge, Random Forest, XGBoost) are evaluated using recursive feature elimination where applicable, with three validation schemes (5-fold cross-validation, spatial leave-one-management-out, and temporal holdout). The workflow concludes with model selection, performance evaluation, prediction of biochemical traits (CP, AMLS, AMLG, LysP, LysG, SC, CF), and downstream interpretation to quantify phenotypic variation and identify genotype responses.

UAV data collection was performed by the Taylor Geospatial Institute (TGI), which conducted flights using RGB and multispectral sensors to acquire high-resolution imagery. Missions were planned and executed using standard flight-planning software, and the UAV followed pre-programmed flight paths to ensure consistent image capture throughout key growth stages in both years (**Supplementary Table S2**). The integration of onboard sensors and precise GPS calibration enabled accurate georeferencing of the imagery, supporting detailed spatial analysis of crop traits. Our team then processed these datasets using the image-processing pipeline we are developing as part of the FieldDock project to generate orthophotos and compute plant indices (Alphabetical List of Spectral Indices, n.d; De Swaef et al., 2021; Gitelson et al., 2003; Grbović et al., 2025; Kataoka et al., 2004; Louhaichi et al., 2001; Memon et al., 2025; Radócz et al., 2023; Reudenbach, 2019; Shammi et al., 2024; List of available Indices, n.d.; rgb_indices function - RDocumentation, n.d) (**Supplementary Tables S3**, **S4**), essential for downstream phenotypic analyses and crop modeling (**Figure 3**). FieldDock is a fully autonomous, end-to-end phenotyping platform using a custom built hexacopter equipped with a MicaSense multispectral camera and capable of both UAV data collection and processing. Its workflows are designed to standardize and scale high-throughput field imaging to revolutionize the way we approach agriculture, making it more efficient, sustainable, and intelligent (About- FieldDock Research Project, n.d.).

#### 2.2.6 Statistical data analysis

A comprehensive statistical pipeline has been implemented to evaluate the effects of genotype, treatment, and year on key variables (e.g., CP, AMLS, AMLG, LysP, LysG, SC, and CF). ANOVA was conducted using R (R version 4.5.1) to analyze the main effects and interactions among the factors, ensuring a detailed assessment of their significance (The R Project for Statistical Computing, n.d.). To validate the assumptions of normality and homogeneity, the Shapiro-Wilk test (González-Estrada et al., 2022) and Levene’s test (Keyes and Levy, 1997) were applied, ensuring the reliability of the statistical inferences drawn from the analysis. We based all inferences on Welch’s ANOVA, which relaxes the equal-variance assumption (**Supplementary Table S5**).

#### 2.2.7 Statistical and machine learning modeling

The statistical and machine learning modeling approach implemented here aims to estimate the target variable, such as CP, AMLS, AMLG, LysP, LysG, SC, and CF, using a robust preprocessing, feature selection, and model evaluation pipeline (**Table 2**). The data are initially cleaned by handling missing values, target values are dropped, and feature columns are imputed with their respective mean. The dataset is split into training and testing subsets based on the year: 2023 for training and 2024 for testing, ensuring temporal separation to simulate real-world predictive scenarios and named temporal validation. This is followed by two complementary validation schemes: (i) 5-fold cross-validation and (ii) spatial validation via leave-one-management-out (eight folds, one per management). This often results in higher accuracy, though it is not necessarily indicative of better real-world performance, as cross-validation does not preserve temporal separation and therefore might overestimate predictive ability in future scenarios. This comprehensive approach enables model comparison to identify the best-performing model for the given data.

**Table 2:** Overview of models and settings used in grain quality estimation.

Model	Library/Class	Key hyperparameters/settings	Feature selection	Standardization	Notes
PLS	sklearn.cross_decomposition.PLSRegression	n_components=2	RFE → 10 features	Yes (StandardScaler)	Linear regression-based, handles multicollinearity, used with RFE.
Lasso	sklearn.linear_model.Lasso	alpha=0.1, random_state=42	RFE → 10 features	Yes	Adds L1 penalty for feature selection.
ElasticNet	sklearn.linear_model.ElasticNet	alpha=0.1, l1_ratio=0.5, random_state=42	RFE → 10 features	Yes	Combines L1 (Lasso) and L2 (Ridge).
Bayesian Ridge	sklearn.linear_model.BayesianRidge	Default (n_iter=300, tol=1e-3, alpha_1 = 1e-6, alpha_2 = 1e-6)	RFE → 10 features	Yes	Probabilistic regression with priors, very stable.
Random Forest	sklearn.ensemble.RandomForestRegressor	n_estimators=100, random_state=42	None	Yes	Tree-based ensemble, good for nonlinear features.
XGBoost	xgboost.XGBRegressor	n_estimators=100, random_state=42, verbosity=0	None	Yes	Gradient boosting, optimized trees, robust to noise.

The pipeline employs a variety of regression models, including PLS, LASSO, ElasticNet, Bayesian Ridge, Random Forest, and XGBoost. The model was tuned using 5-fold cross-validation to determine the optimal number of PLS components. Feature standardization is performed using StandardScaler to ensure models operate on comparable scales, with scaling parameters learned from the training set and subsequently applied to the test set to avoid data leakage. Recursive Feature Elimination (RFE) is applied within each cross-validation fold, using each linear model (PLS, LASSO, ElasticNet, and Bayesian Ridge) as its own base estimator and a step size of 1 to iteratively remove the lowest-ranked features until 10 remain (**Table 2**). RFE was also applied to LASSO and ElasticNet, despite their embedded feature-selection capability, to maintain a consistent feature-selection framework across all linear models, enabling fair comparison of model performance and SHAP-based interpretability. For Random Forest and XGBoost, all hyperparameters other than the number of trees and random seed were left at their library defaults. This choice was intentional, as the small sample size makes extensive hyperparameter tuning prone to overfitting, and using defaults ensures comparability across models while reducing the risk of overly optimistic performance estimates. Each model’s performance is assessed using Root Mean Square Error (RMSE) and the coefficient of determination (R²) score on unseen test data. The model’s performance is visualized through scatter plots of observed vs. predicted traits, highlighting the comparative accuracy of each model.

## 3 Results

### 3.1 Weather conditions

**Figure 4:**
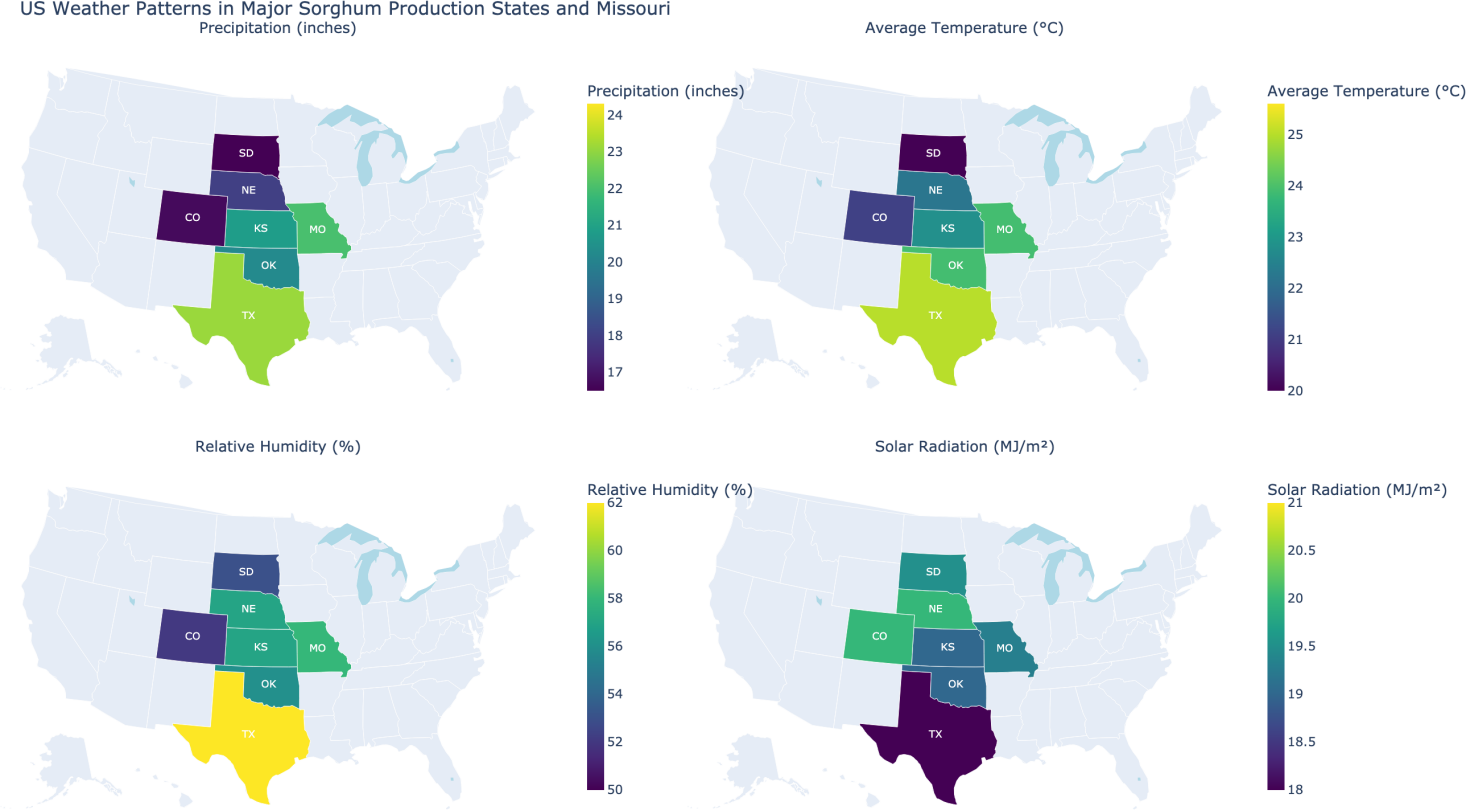
US weather patterns in major sorghum production states and Missouri in 2023 and 2024. This figure presents four choropleth maps highlighting key weather variables across major sorghum-producing regions in the United States and Missouri (experiment site). Each map focuses on a different variable for the 2023 and 2024 agricultural seasons, such as precipitation cumulus (inches), average temperature (°C), relative humidity (%), and total solar radiation (MJ/m²), using color gradients to indicate the relative intensity or amount of each trait within different states.

We observed distinct regional variations in weather conditions that influence sorghum growth in the United States (**Figure 4**). States in the southern and southeastern areas (e.g., Texas) tend to show higher temperatures and humidity levels, while parts of the central plains (e.g., Nebraska) often exhibit higher solar radiation and moderate humidity. These climatic differences can play a significant role in determining optimal sorghum cultivation strategies and potential yields across these regions. Among these states, Missouri stands out for its particularly favorable balance of precipitation, temperature, and humidity, combined with adequate solar radiation. This represents a favorable climatic profile for sorghum cultivation and was observed during our field experiments in Saint Charles (**Supplementary Figure S1**).

During the June to October period in Saint Charles, MO (2023 and 2024), the daily temperature showed a pronounced midday peak, while relative humidity exhibited an inverse pattern, generally reaching its highest levels in the early morning or late evening (**Supplementary Figure S1**). Solar radiation peaked around noon, reflecting maximum sun intensity at midday. Although the two years followed similar diurnal cycles, minor differences in temperature and humidity amplitude point to year-to-year variability. These findings provide a clear snapshot of the local weather conditions under which the field experiment was conducted, illustrating how temperature, humidity, and solar radiation evolve throughout the day in this region.

### 3.2 Effect of management practices on plant photosynthesis and yield

**Figure 5:**
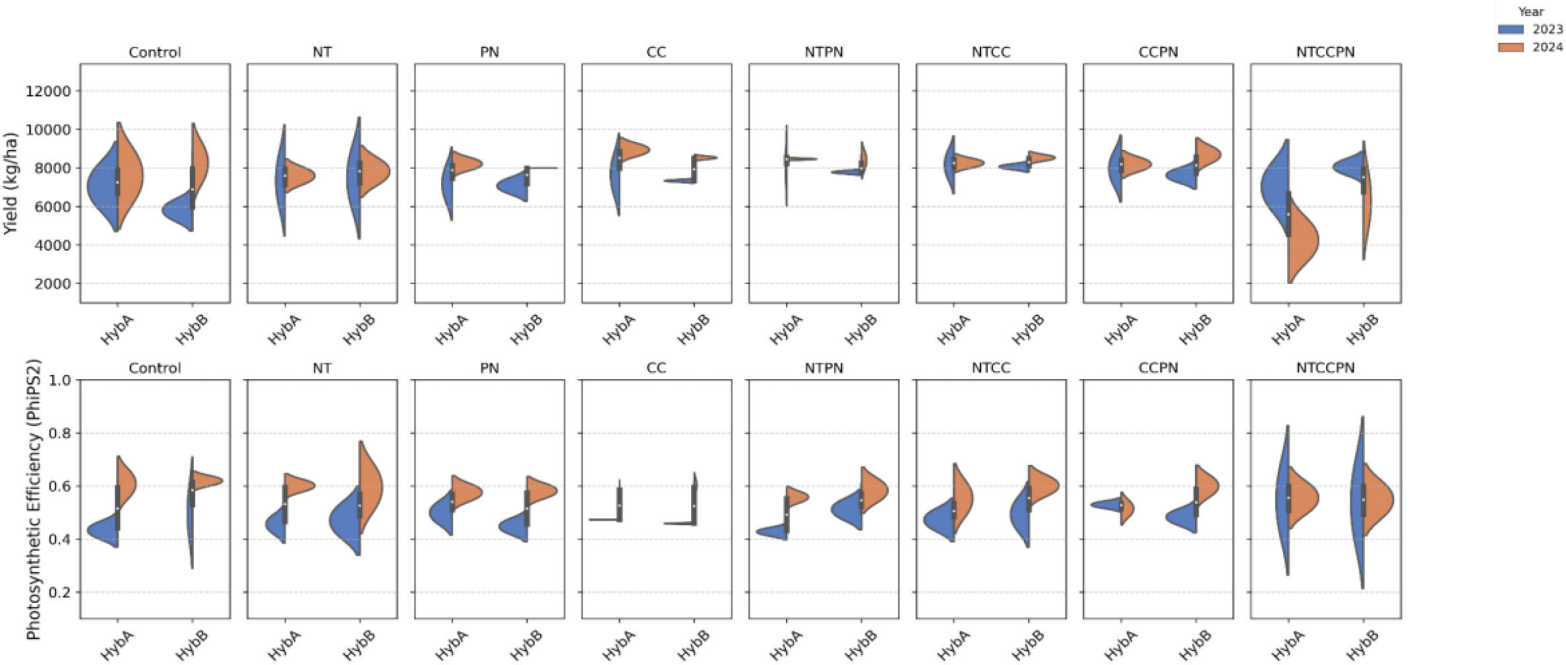
Comparative trait performance of sorghum hybrids under diverse management strategies. The top panel of violin plots shows the distribution of yield (kg/ha) and the second panel displays the photosynthetic efficiency, PhiPS2 in sorghum hybrids (Hybrid A in blue, and Hybrid B in orange) across eight management practices (Control, NT, PN, CC, NT, NTPN, NTCC, CCPN, and NTCCPN).

The violin plots (**Figure 5**) reveal distinct physiological and yield responses between sorghum hybrids HybA and HybB across eight management practices. HybB demonstrates more consistent yield performance, particularly under PN, CC, NTPN, and NTCC treatments for both years 2023 and 2024, as evidenced by tighter distribution patterns. In contrast, HybA shows greater stability across these management practices. The photosynthetic efficiency (PhiPS2) reveals variation for both years, particularly under NTCCPN. HybA demonstrates broader distribution, suggesting greater sensitivity or adaptability. We observed variation in yield and PhiPS2 in both years under Control, PN, CC, and NTPN for both hybrids.

The management practices appear to influence the physiological traits differently across hybrids, suggesting that the interaction between hybrid type and management approach could play a critical role in optimizing both physiological performance and yield.

### 3.3 Grain composition trait analyses

#### 3.3.1 Temporal dynamics of sorghum grain quality trait responses under varying management practices

**Figure 6:**
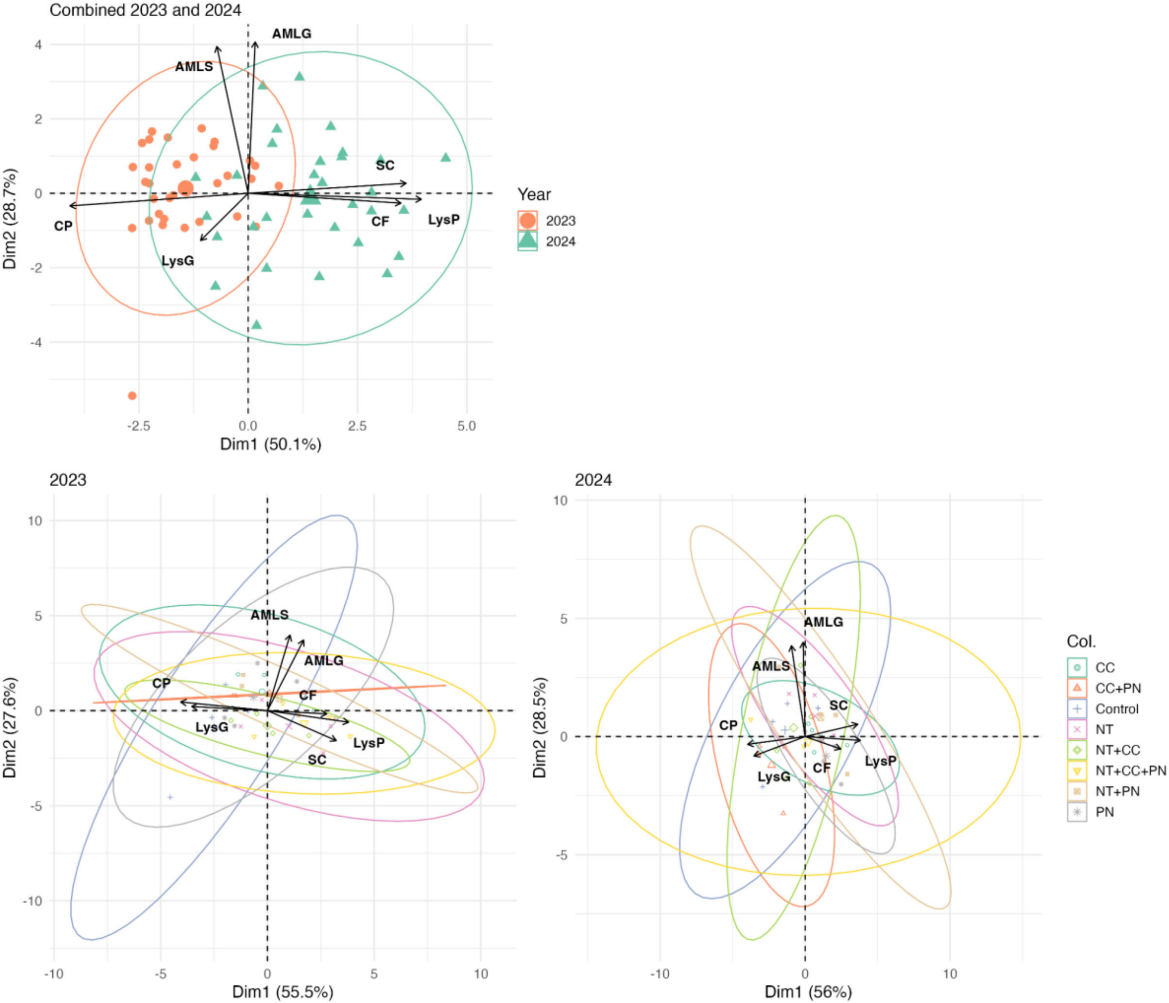
Multivariate analysis of sorghum traits: yearly and treatment-based clustering. This figure presents a principal component analysis (PCA) biplot illustrating the variation in sorghum seed composition traits across different years (2023 and 2024) and treatment groups. The top panel shows the combined PCA results for both years, while the bottom panels depict individual PCA analyses for 2023 and 2024, highlighting treatment-based clustering with confidence ellipses.

The principal components (Dimension 1 and Dimension 2) explain 50.1% and 28.7% of the total variance, respectively, in the combined 2023 and 2024 dataset, with individual year analyses revealing similar trait clustering patterns (**Figure 6**). The genotypes from 2023 (orange circles) and 2024 (green triangles) form distinct clusters, emphasizing the influence of year and environmental conditions on trait variation. Traits such as LysP, SC, and CF strongly align along Dimension 1, indicating their key role in differentiating genotypes in the 2024 season, while CP was the most involved in genotypes’ differentiation in 2023. Nevertheless, AMLG, and AMLS appeared to be the more stable traits across 2023 and 2024.

The treatment-based clustering shown separately for 2023 and 2024 further reveals that management practices contribute to genotype performance variations, with some treatments (NTCCPN) enhancing stability across years. The overlapping and non-overlapping regions of the ellipses highlight both consistent (Control) and dynamic responses (NTCC) of genotypes under different conditions. This analysis underscores the necessity of multi-environmental and multi-management trials to capture trait variability and identify resilient sorghum genotypes for improved grain quality.

#### 3.3.2 Effect of management practices on key grain biochemical traits in two sorghum hybrids

**Figure 7:**
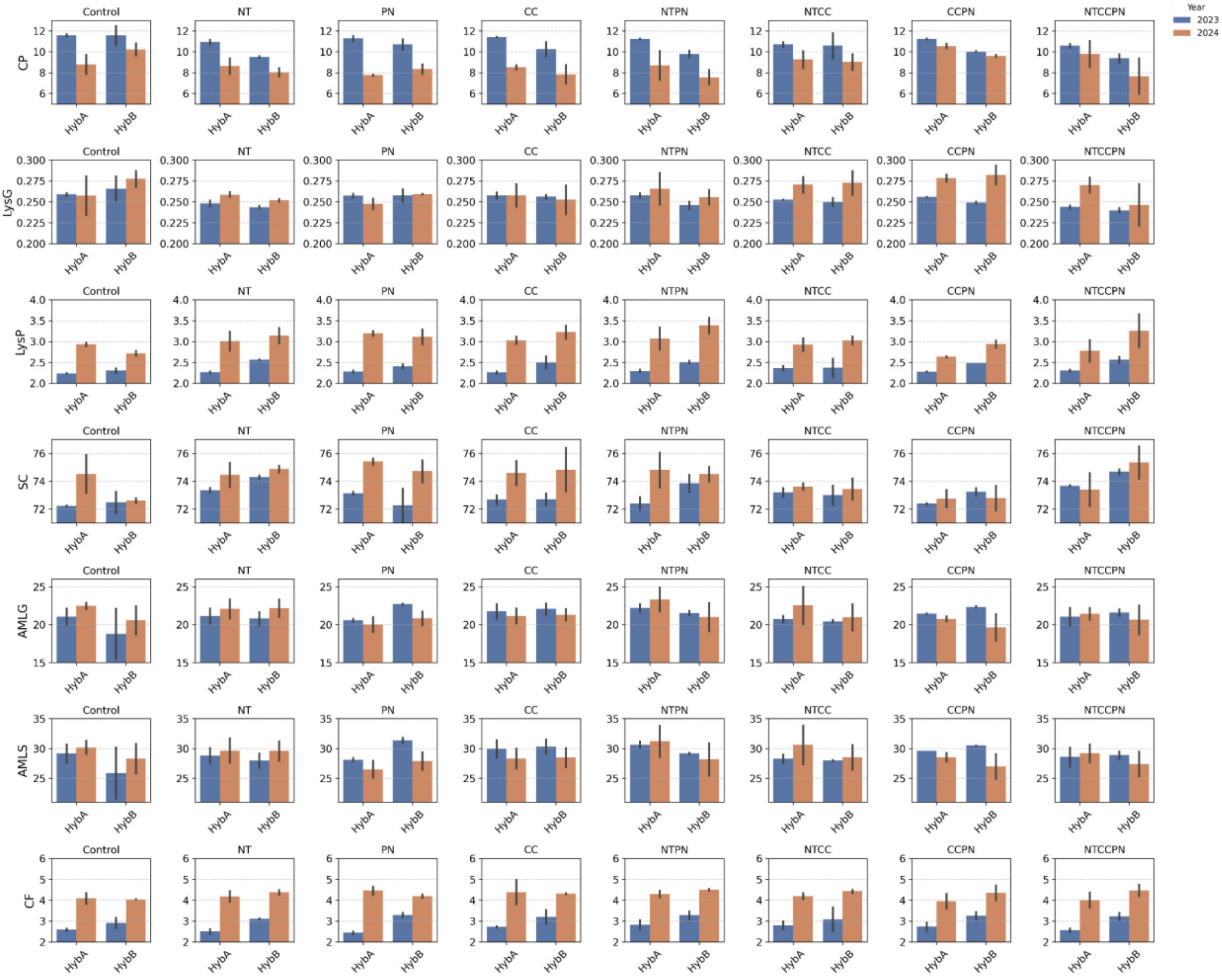
Comparison of grain quality traits of two sorghum hybrids grown under various management practices in 2023 and 2024.

The statistical summary of the significance of genotype, treatment, year and their interactions is presented in **Supplementary Table S5**, showing the heteroscedasticity-robust ANOVA (Welch) results for genotype, treatment, and year effects across traits. All traits show strong treatment, year, and treatment × year interactions effects, while Amylose (AMLS and AMLG) and LysP also exhibited significant genotype and treatment interaction effects. These findings highlight that optimizing grain nutritional quality in sorghum requires genotype-specific management strategies. To further support the interpretation of these interaction patterns, we additionally performed Games–Howell *post-hoc* multiple comparisons to test genotype differences within each management × year combination, and these results are reported in **Supplementary Table S6**. A detailed variation in these key grain biochemical traits, for the two commercial sorghum hybrids (HybA and HybB) under different management practices, including Control, No-Till, Precision Nitrogen (PN), Cover Crop (CC), and their combinations, is presented in **Figure 7** as a descriptive summary of treatment patterns. This result suggests that farmers might choose HybA for higher crude protein content under CC, No Till + PN, CC + PN, or NTCCPN combinations, whereas HybB could be preferred for higher lysine content in protein under CC, No Till + PN, CC + PN, or No Till + CC + PN managements. These insights can guide breeders and agronomists in selecting appropriate hybrids and management practices to enhance the nutritional quality of sorghum.

### 3.4 Grain compositional traits modeling: relationship between observed and predicted traits

#### 3.4.1 Crude protein

**Figure 8:**
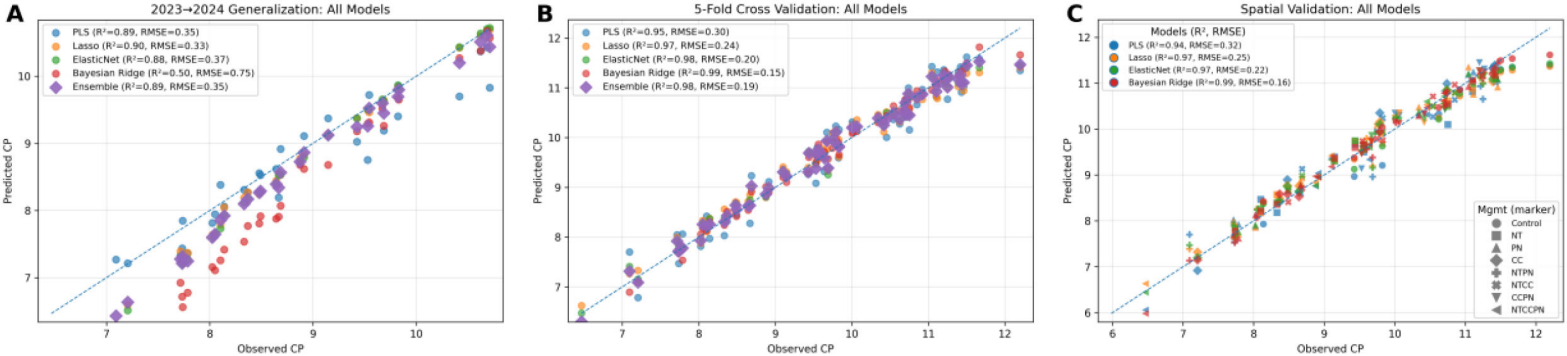
Regression plots of observed vs. predicted crude protein content (CP) using machine learning models. The dashed diagonal represents the 1:1 relationship between observed and predicted crude protein content (CP). Six machine learning models are evaluated: PLS, LASSO, ElasticNet, Bayesian Ridge, XGBoost, and Random Forest using temporal **(A)**, cross **(B)**, and spatial **(C)** validation methods. The dots indicate individual data points, with their proximity to the diagonal line reflecting prediction accuracy. Each subplot displays two model performance metrics: the coefficient of determination (R²) and Root Mean Squared Error (RMSE).

**Figure 8** illustrates observed and predicted crude protein (CP) across genotypes and treatments. Six machine learning models were evaluated in this study: Partial Least Squares (PLS), LASSO, Elastic Net, Bayesian Ridge, Random Forest (RF), and XGBoost. The figure highlights the three models with the best performance, LASSO, Elastic Net, and Bayesian Ridge. These models demonstrated consistent predictive ability, capturing both high and low CP regions across treatments and genotypes. Their strong performance indicates a capacity to generalize effectively, making them suitable for identifying management strategies that enhance protein content. In contrast, the other three models, PLS, RF, and XGBoost, underperformed, likely due to overfitting or limited ability to represent complex nonlinear relationships between traits and environment.

#### 3.4.2 Amylose from starch and grain

**Figure 9:**
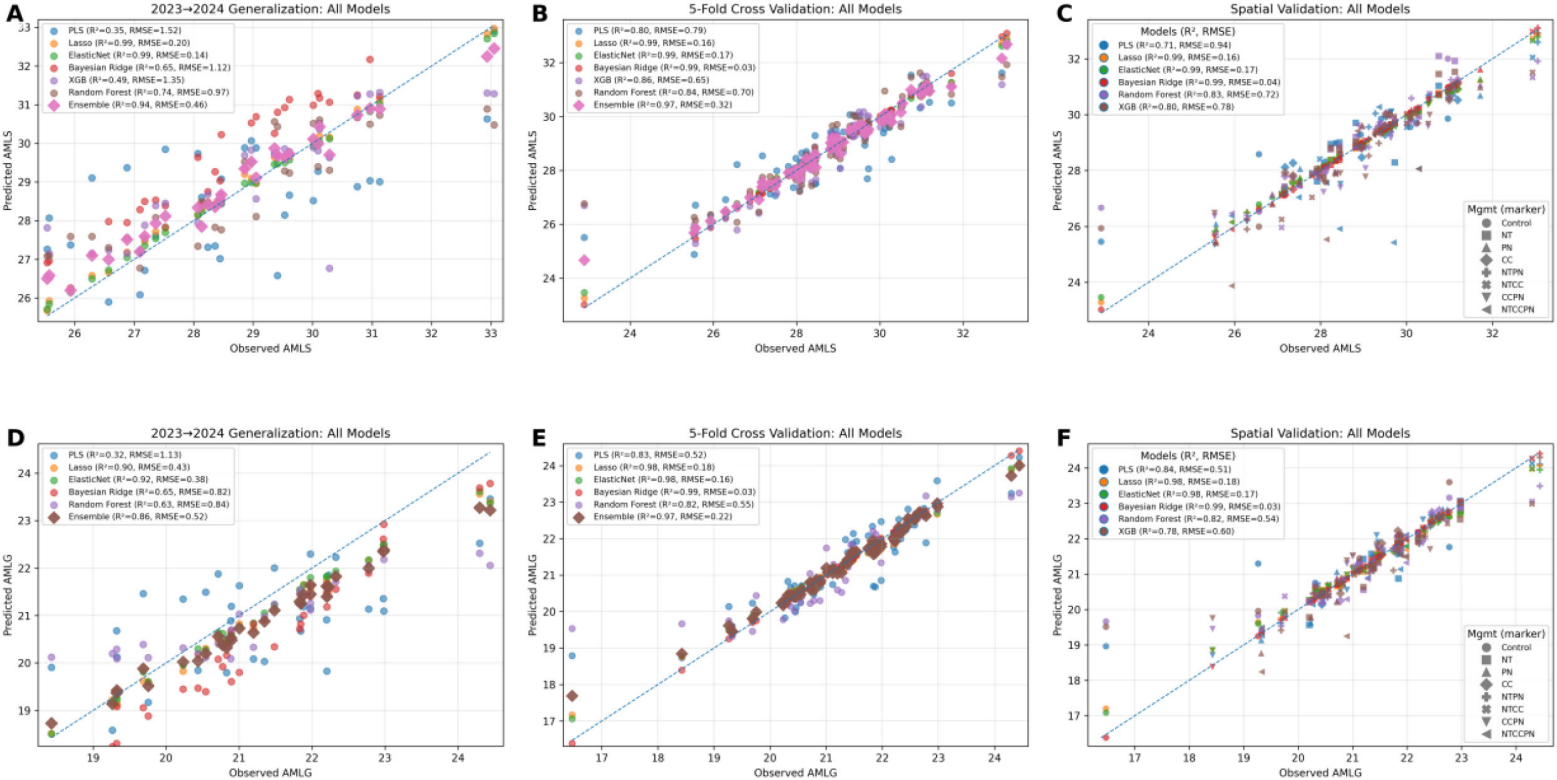
Regression plots of observed vs. predicted amylose in starch (AMLS) and grain (AMLG) content using PLS, LASSO, ElasticNet, Bayesian Ridge, XGBoost, and Random Forest models with temporal **(A, D)**, cross **(B, E)**, and spatial **(C, F)** validations methods.

Across six machine-learning models (PLS, LASSO, ElasticNet, Bayesian Ridge, XGBoost, and Random Forest), we achieved strong prediction performance for amylose in starch (AMLS) and generally lower for amylose in grain (AMLG) (**Figure 9**). For AMLS, LASSO and ElasticNet were top performers, each reaching R² ≈ 0.99 with low RMSEs (≈0.20 and 0.14), likely because their regularization and feature selection handle multicollinearity and highlight the most informative predictors for this trait. XGB showed moderate success (R² ≈ 0.49) with noticeable over-/underestimation consistent with sensitivity to noise and interaction effects that can induce overfitting, while PLS had the lowest R² (≈0.35), underscoring the value of explicit feature selection. For AMLG, five of the six models performed successfully (excluding XGB), but overall accuracy tended lower than for AMLS, suggesting that grain-level amylose is influenced by broader compositional, genetic, and environmental factors. Notably, ElasticNet and LASSO were consistently strong across both traits, indicating good generalization and making them promising candidates for deployment in breeding pipelines where robustness across biological contexts is essential.

#### 3.4.3 Lysine from protein and starch content

**Figure 10:**
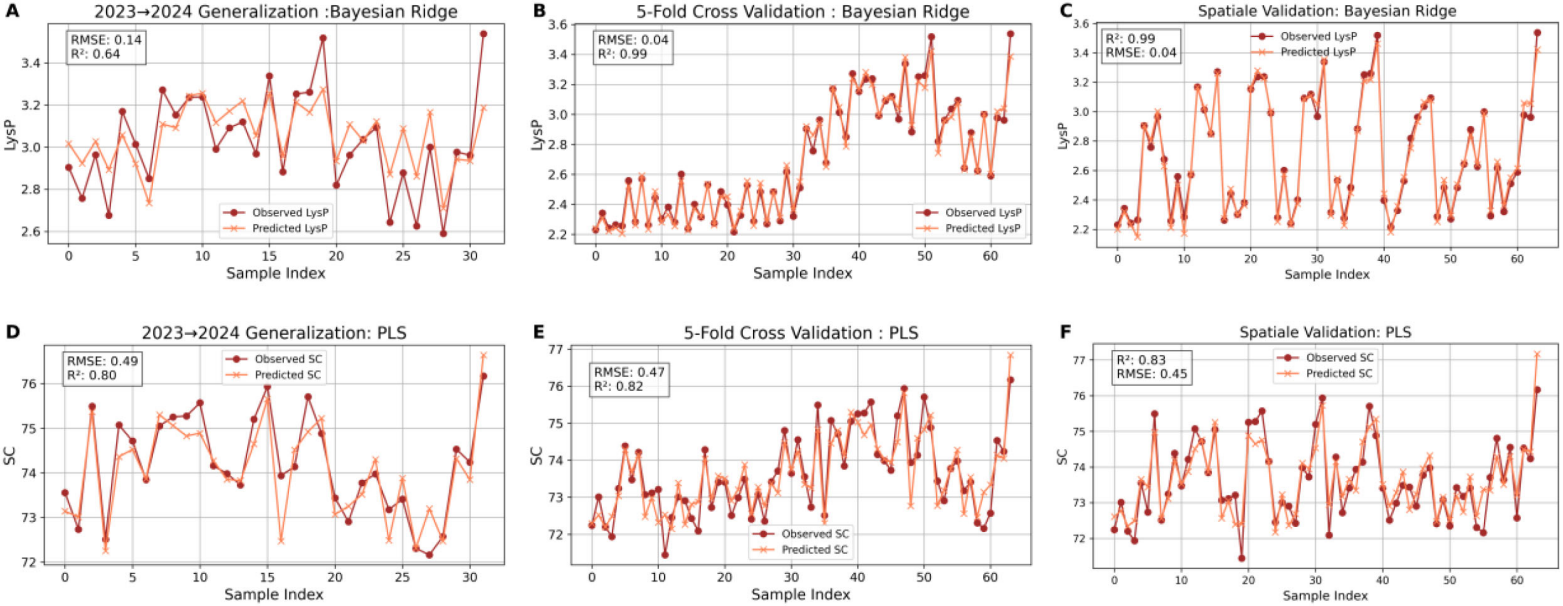
Relationship of observed vs. predicted values for lysine (LysP) and starch content (SC) in sorghum samples using PLS, and Bayesian Ridge models. The first row of the graph shows lysine content on a protein basis (LysP) predictions using the Bayesian Ridge model, with observed values (dark red circles) and predicted values (light orange crosses). The second row of the graph presents starch content (SC) predictions using the PLS model. **(A, D)** temporal validation; **(B, E)** cross validation; **(C, F)** spatial validation.

The performance of predictive models when evaluating two traits: lysine content on a protein basis (LysP) and starch content (SC) using 2023 and 2024 data is presented in **Figure 10**. For LysP, the Bayesian Ridge model prediction achieved an RMSE of 0.14 and an R² of 0.64. Meanwhile, the starch content predictions using the PLS model achieved an RMSE of 0.49 and an R² of 0.80, suggesting a strong correlation between predicted and actual values. Among the six models tested, only the Bayesian Ridge model for LysP and the PLS model for SC demonstrated significant predictive capability for the 2024 dataset. The superior performance of these models highlights their suitability for predicting these specific traits, making them critical for advancing trait-based modeling and selection strategies.

#### 3.4.4 Cross-validation success vs. real-world struggles: performance gaps in predicting lysine from grain and crude fat

**Figure 11:**
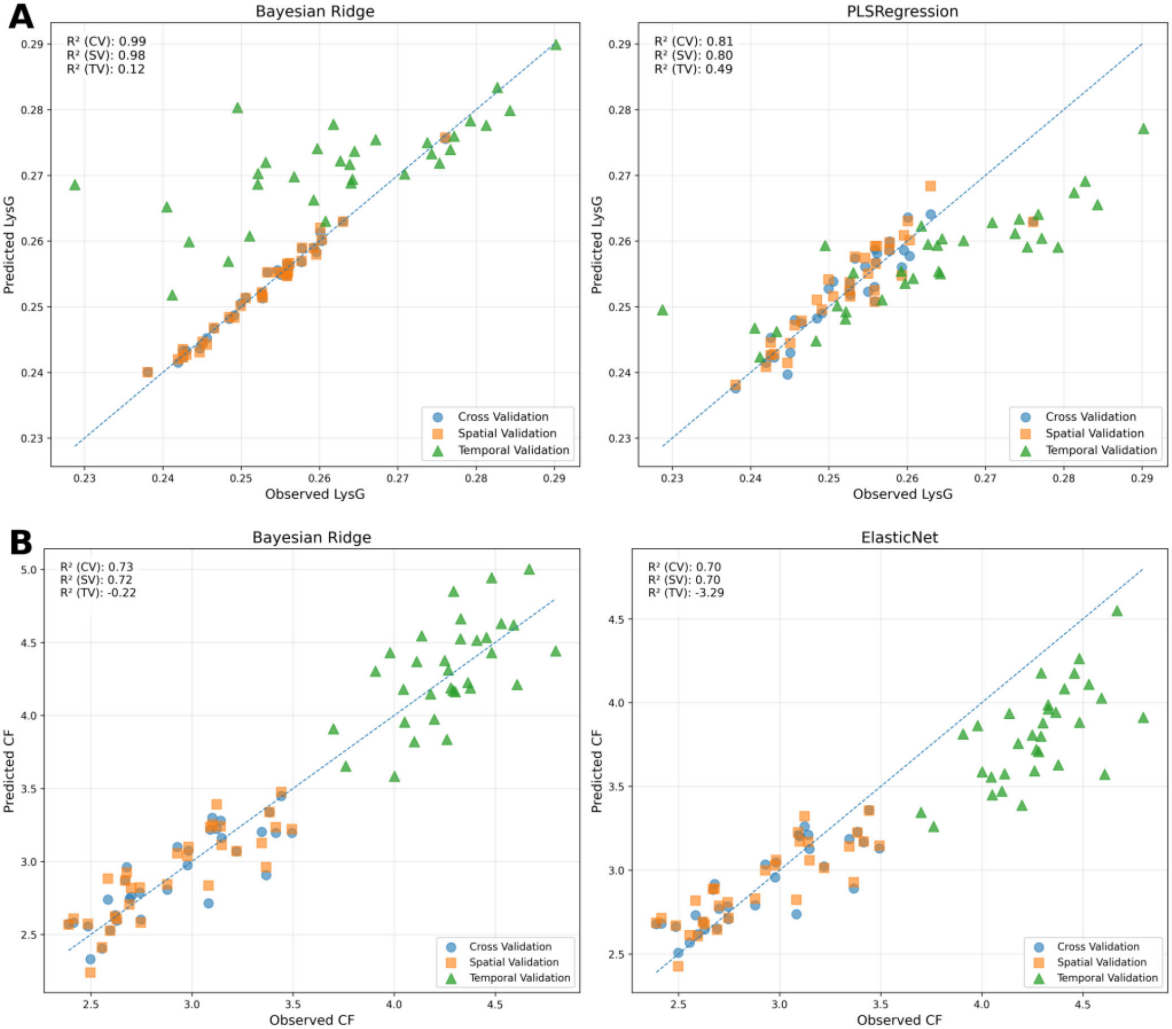
Relationship between observed and predicted lysine (LysG) and crude fat (CF) from temporal (TV) **(A)**, spatial (SV) **(B)**, and cross-validation (CV) techniques using ML models. Scatter plots comparing observed and predicted lysine on a grain basis (LysG) and crude fat (CF) content using Bayesian Ridge and PLS; Bayesian Ridge and ElasticNet regression models respectively. The dashed diagonal line indicates perfect prediction (1:1 relationship). Performance metrics include R² for both for all validation methods.

The relationship between observed and predicted values for lysine (LysG) and crude fat (CF) using different models and evaluation techniques highlights the performance discrepancy between cross-validation and temporal validation methods (**Figure 11**). For LysG content, both Bayesian Ridge and PLS regression models demonstrate robust performance under cross-validation (R² = 0.98 and 0.82, respectively), making them suitable models for this trait when cross-validation is used.

For crude fat (CF), predictions show good accuracy scores with cross-validation, using Bayesian Ridge and ElasticNet models, achieving R² values of 0.74 and 0.77. However, when evaluating these models on the 2024 dataset (training on 2023 data and testing on 2024 data named temporal validation), all models show a significant performance drop. For example, the Bayesian Ridge model’s predictive power for LysG drops to an R² of 0.12, while CF predictions show negative R² values (R² = -0.22 and -3.39 for Bayesian Ridge and ElasticNet, respectively). This comparison highlights the challenge of predicting traits accurately in the 2024 dataset, emphasizing the potential effects of temporal or environmental variability. The figures effectively demonstrate the contrast between high performance during cross-validation and the difficulty in generalizing to future datasets when using temporal validation.

### 3.5 Comparative analysis of the different models

**Figure 12:**
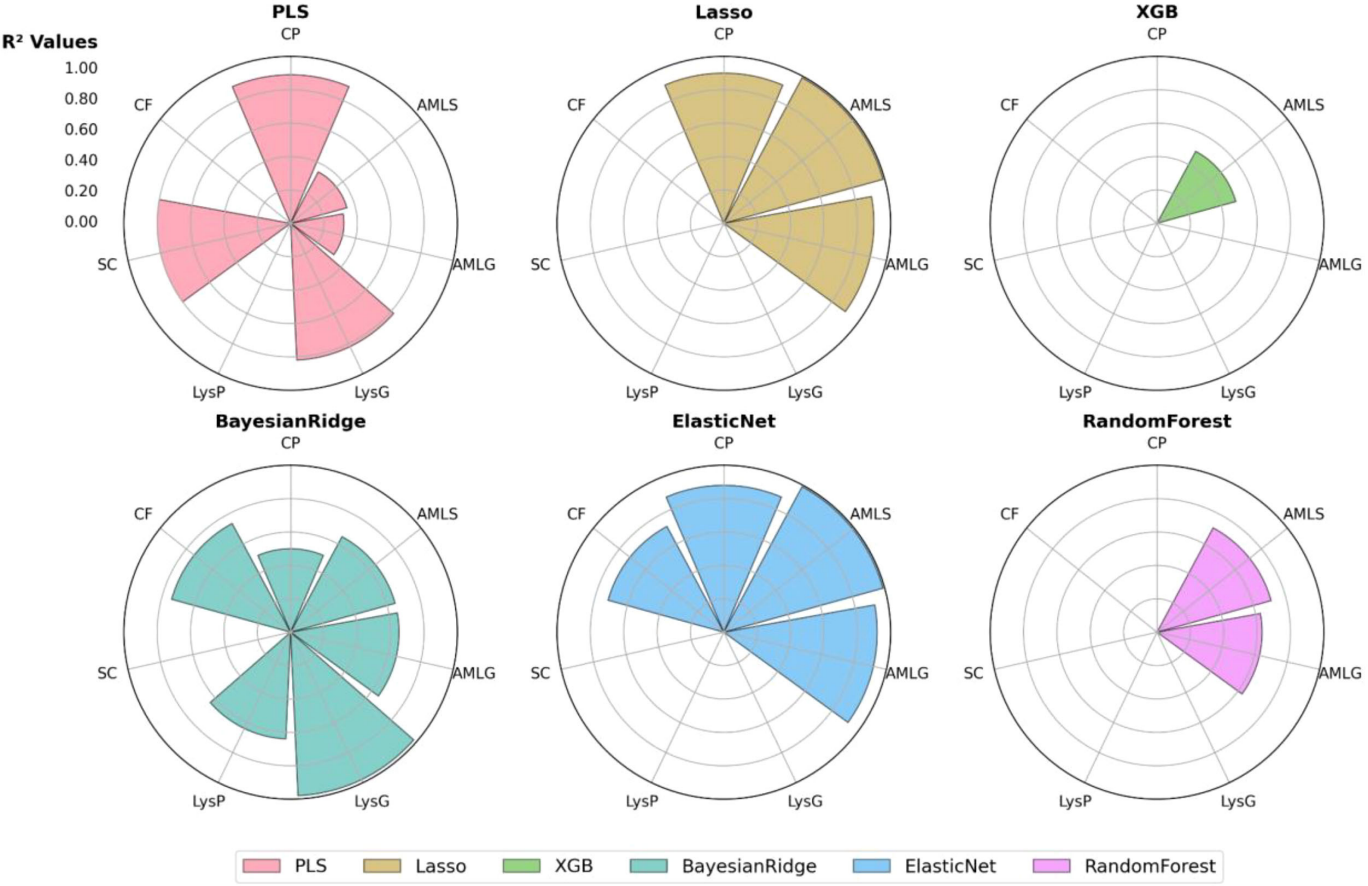
Comparative performance analysis of machine learning model across biochemicals sorghum grain traits. These radar plots compare six machine learning models’ predictions (PLS-light pink, Bayesian Ridge-cyan, LASSO-light brown, ElasticNet-blue, XGBoost-green, and Random Forest-purple) across seven biochemical traits (LysG, LysP, SC, CF, CP, AMLS, and AMLG). Left values show R² values (0–1 scale), where larger colored polygons indicate better model fit.

The radar charts (**Figure 12**) provide a comparative analysis of model performance across multiple grain biochemical traits based on the R² metric (goodness of fit). Higher R² values indicate better model performance in explaining trait variability, while lower values suggest weaker predictive capacity. Models such as PLS and Bayesian Ridge are broad-spectrum, showing effectiveness across many traits with high or moderate R² values, whereas models like XGB or RF appeared to be dedicated predictors for AMLS. LASSO and ElasticNet acted as trait-cluster models for amylose and protein. These differences arise because each trait exhibits a distinct correlation structure and signal-to-noise profile, making certain algorithms better suited to specific biochemical properties. For example, traits with strong linear relationships and correlated predictors, such as crude protein and amylose, favor regularized linear models, while traits influenced by more complex multicollinearity patterns, like starch content, benefit from dimensionality-reduction approaches such as PLS.

The differences in model performance underscore the varying complexity of trait estimation, with regularization-based models (LASSO, ElasticNet, Bayesian Ridge) showing greater robustness in handling trait variability. Meanwhile, tree-based models like Random Forest and XGB exhibit inconsistent performance across traits, excelling in some while underperforming in others. These insights suggest that selecting the optimal model depends on the trait of interest, and **Table 3** provides guidance on model selection.

**Table 3:** Guidance on model choice in sorghum grain biochemical composition traits modeling.

Trait	Abbreviation	Model	Technique		Score	
			Train	Test	R^2^	RMSE
Crude Protein	CP	LASSO	2023	2024	0.9	3.39
Amylose from Starch	AMLS	ElasticNet	2023	2024	0.99	0.48
Amylose from Grain	AMLG	ElasticNet	2023	2024	0.92	1.78
Lysine from Protein	LysP	Bayesian Ridge	2023	2024	0.64	5.18
Starch	SC	PLS	2023	2024	0.8	0.66
Lysine from Grain	LysG	Bayesian Ridge	2023	2024	0.12	4
Crude Fat	CF	Bayesian Ridge	2023	2024	-0.22	7.5
Crude Protein	CP	LASSO	Cross-Validation		0.97	0.24
Amylose from Starch	AMLS	ElasticNet	Cross-Validation		0.99	0.16
Amylose from Grain	AMLG	ElasticNet	Cross-Validation		0.98	0.16
Lysine from Protein	LysP	Bayesian Ridge	Cross-Validation		0.99	0.04
Starch	SC	PLS	Cross-Validation		0.82	0.47
Lysine from Grain	LysG	Bayesian Ridge	Cross-Validation		0.98	1.32
Crude Fat	CF	Bayesian Ridge	Cross-Validation		0.74	4.53
Crude Protein	CP	LASSO	Spatial Validation		0.97	0.25
Amylose from Starch	AMLS	ElasticNet	Spatial Validation		0.99	0.17
Amylose from Grain	AMLG	ElasticNet	Spatial Validation		0.98	0.17
Lysine from Protein	LysP	Bayesian Ridge	Spatial Validation		0.99	0.04
Starch	SC	PLS	Spatial Validation		0.83	0.45
Lysine from Grain	LysG	Bayesian Ridge	Spatial Validation		0.88	0.004
Crude Fat	CF	Bayesian Ridge	Spatial Validation		0.91	0.22

This table shows each trait and the most performant model as well as accuracy score and technique used.

### 3.6 Important feature in predicting grain biochemical composition traits

**Figure 13:**
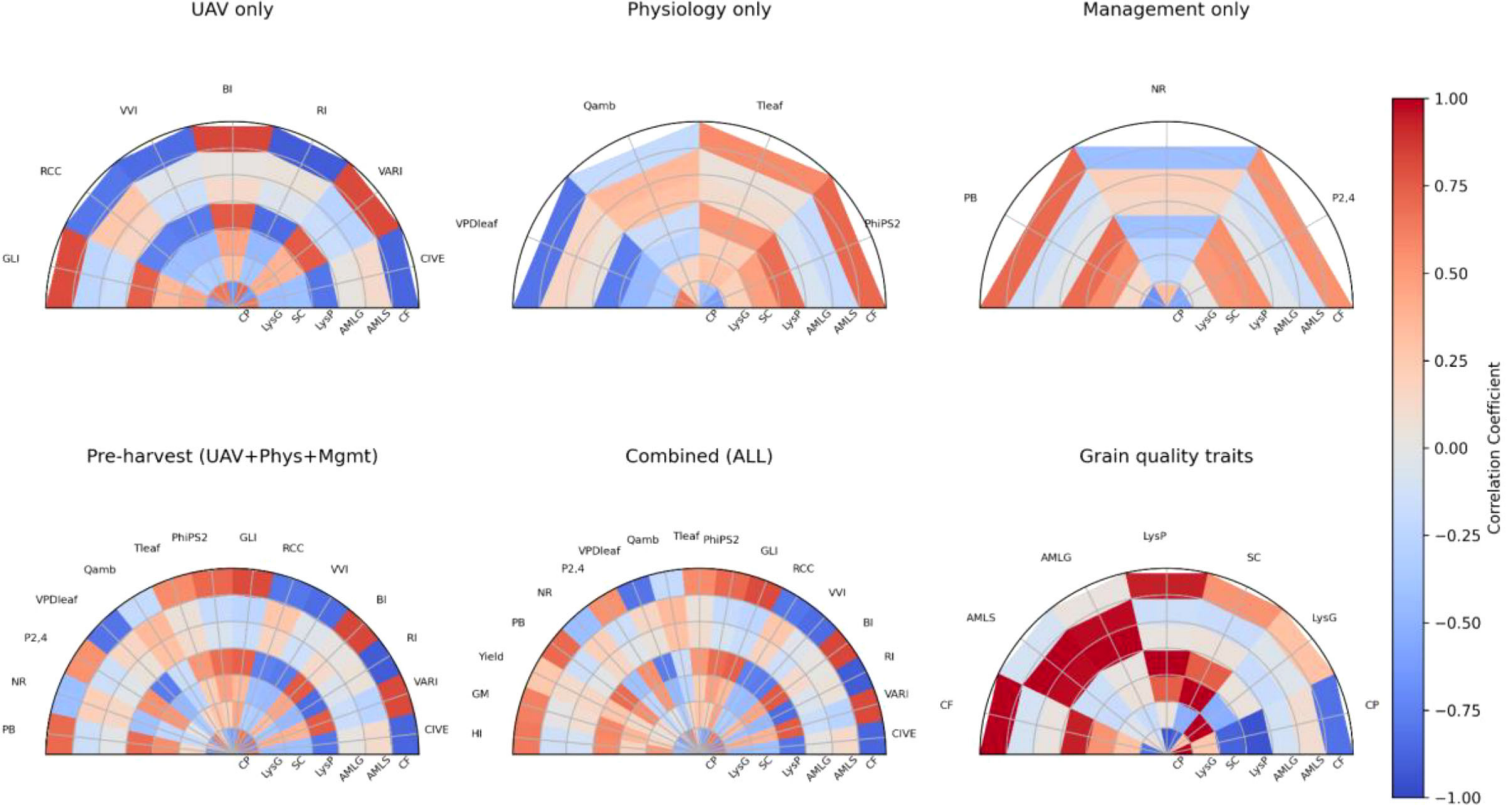
Feature group ablation and correlation with grain quality traits. Half-circle correlation plots show the relationships between grain quality traits (radial axis) and six feature sets: UAV only, Physiology only, Management only, Pre-harvest (UAV + Physiology + Management), and Combined (ALL) (includes harvest variables such as yield and harvest index). A final panel shows pairwise correlations among the grain quality traits for reference. GM, grain moisture; HI, harvest index; GLI, Green Leaf Index; VARI, Visible Atmospherically Resistant Index; CIVE, Color Index of Vegetation Extraction; VVI, Visible Vegetation Index, Tleaf, leaf temperature, VPDleaf, vapor pressure deficit, gsw, stomatal conductance, Qamb, ambient light.

We performed a feature group ablation (UAV-only, physiology-only, management-only, pre-harvest only, and all-available except target) to evaluate the marginal predictive utility of each group and to identify if accurate models can be built with a smaller, less costly set of assays. **Figure 13** provides valuable insights into relationships between different trait categories, highlighting important features that influence model prediction. For instance, in the “Physiology vs Grain Composition” heatmap, PhiPS2 demonstrates strong positive correlations with LysP (0.69) and CF (0.71), suggesting its potential role as a predictor for LysP and CF in grain. Similarly, VPDleaf shows a notable negative correlation with crude fat (-0.81) while demonstrating a positive correlation with CP (0.64). This indicates that vapor pressure deficit could significantly influence both crude fat and protein levels. These physiological traits appear to be critical in determining biochemical composition of sorghum grain. UAV traits, such as CIVE (Color Index of Vegetation) and GLI (Green Leaf Index), display strong positive correlations with CF (0.87 and 0.81, respectively) and SC (0.74 and 0.43, respectively), highlighting the potential of UAV-derived vegetation indices in assessing grain biochemical composition. These findings underscore the pivotal role of UAV, physiology and pre-harvest traits in estimating specific grain biochemical composition traits particularly crude fat and LysP.

### 3.7 Modeling linear trait interactions for predicting LysP and CF

**Figure 14:**
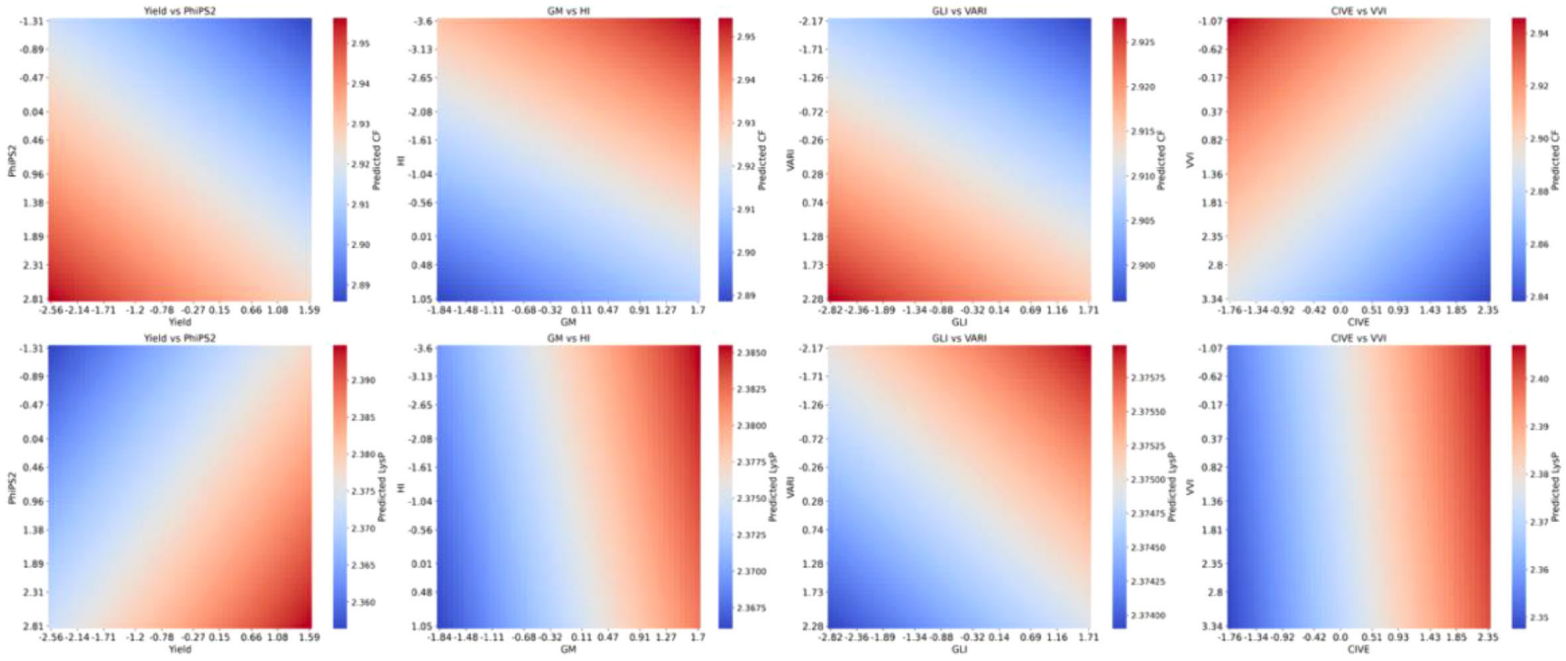
Partial dependence plots of trait interactions for predicting lysine from protein (LysP), and crude fat (CF) content in sorghum grain using Bayesian Ridge Model.

The partial-dependence plots from the Bayesian Ridge models show linear, and additive responses of traits interaction in crude fat (CF) and lysine from protein (LysP) models (**Figure 14**). For LysP, predictions increase with both Yield and PhiPS2, rise with GM, decline with HI, and show higher values with greater GLI and lower VARI. LysP also increases with CIVE but slightly declines as VVI increases. For CF, predictions increase with PhiPS2 and decrease with Yield; they increase with GM but decline with HI. CF is higher at lower GLI and higher VARI, and it decreases as CIVE and VVI increase. The smooth, planar gradients across panels suggest limited interaction or threshold behavior within the observed ranges, supporting an additive interpretation of these effects (**Figure 14**).

### 3.8 Modeling non-linear trait interactions for predicting amylose content in grain and starch.

**Figure 15:**
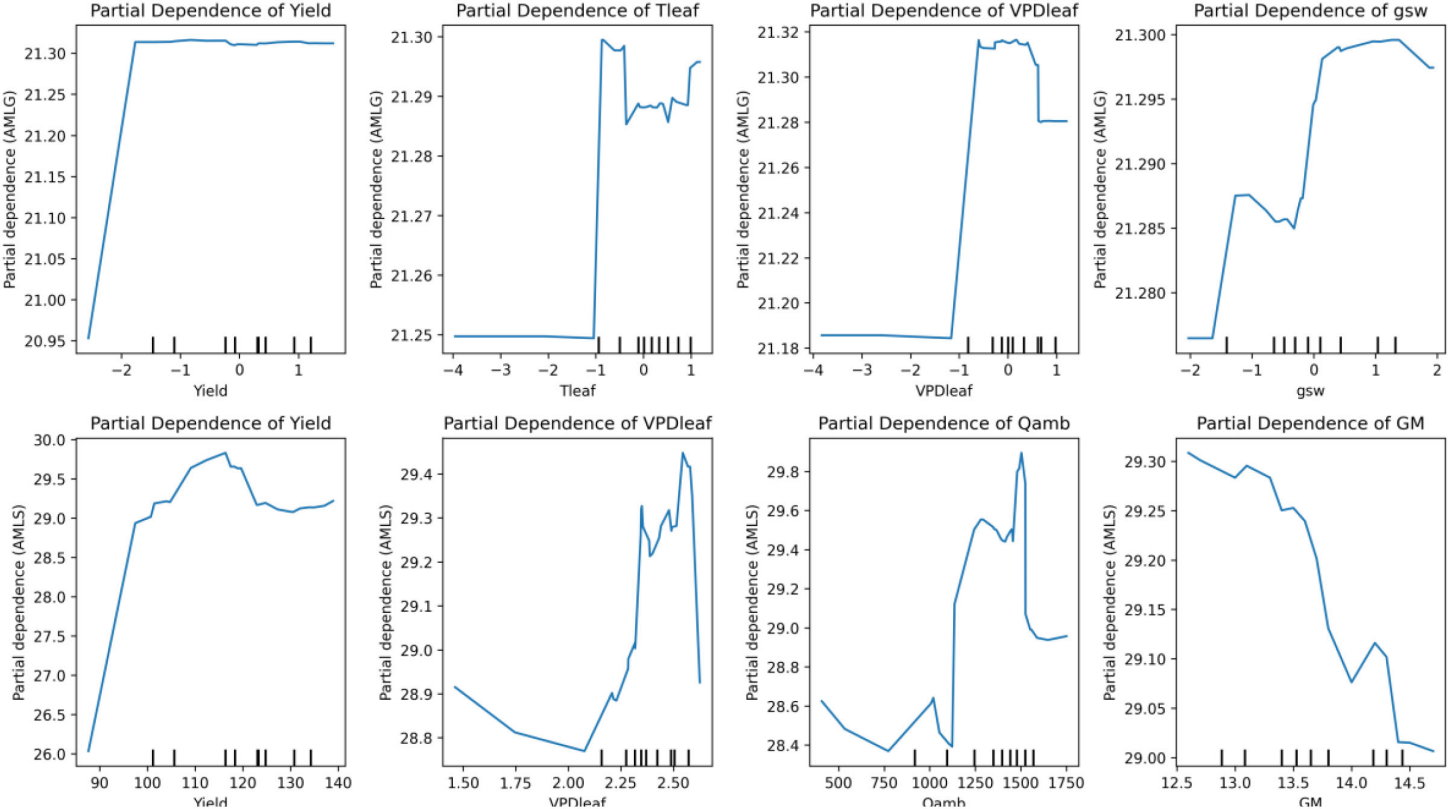
Partial dependence plots of important predictors affecting amylose in grain (AMLG) and starch (AMLS) using Random Forest models.

The partial dependence plots (PDPs) (**Figure 15**) highlight the non-linear relationships between key physiological and environmental traits and their influence on amylose content in grain (AMLG) and starch (AMLS) as predicted by the Random Forest (RF) models. Compared to linear statistical models (PLS, and Bayesian Ridge), which assume linear feature effects, the Random Forest model effectively captures complex and non-linear dependencies, making it suitable for understanding how these important traits contribute to amylose accumulation.

In both the AMLG_RF and AMLS_RF models, traits such as yield, leaf temperature (Tleaf), vapor pressure deficit (VPDleaf), stomatal conductance (gsw), ambient light (Qamb), and grain moisture (GM) emerge as significant predictors, with varying levels of influence across different conditions (**Figure 15**). The PDPs reveal non-linear responses, where abrupt shifts or threshold effects are observed, suggesting that small changes in these traits may lead to significant differences in amylose content. This nonlinear behavior underscores the importance of accounting for complex trait interactions in predicting amylose in starch and grain composition, offering insights that could enhance targeted breeding strategies for crops with optimized amylose content. A more in-depth investigation under greenhouse conditions, in which these traits are varied and their effects on grain composition are measured, offers a promising approach to more precisely track thresholds and sudden shifts.

## 4 Discussion

The findings of this study underscore the potential of integrating machine learning models with multi-environmental field trials and UAV-derived data for predicting key grain biochemical traits in sorghum. By combining predictive modeling, physiological assessments, and management practices, we have provided an enhanced understanding of the complex interactions among genotype, environment, and management that influence sorghum yield and grain quality.

### 4.1 Understanding genotype, and management effects

Our study revealed significant treatment or management effects for all traits, with genotype x treatment interactions significant for traits like Amylose and Lysine, suggesting that optimal management strategies are highly genotype-dependent (Reddy et al., 2003; Hammer et al., 2019). For example, Hybrid A recorded higher crude protein content in CC, NT + PN, CC + PN or NTCCPN, while Hybrid B exhibited superior lysine content under CC, NT + PN, CC + PN, or NT + CC + PN. This indicates that a “one-size-fits-all” approach is ineffective, and tailored strategies are essential for optimizing grain quality (Seyoum et al., 2019), including crude protein and lysine in sorghum plants. These genotype-specific patterns can offer preliminary decision-making guidance, for instance, selecting Hybrid A for production systems targeting higher protein content and Hybrid B for applications emphasizing lysine under the management combinations that consistently enhanced these traits in our study. However, because these responses varied across treatments and years, such recommendations should be treated as provisional until validated across multiple environments. The significant interactions between genotype and year, or treatment and year, further emphasize the dynamic nature of these traits across varying environmental conditions. This variability suggests that environmental factors, such as rainfall patterns and soil moisture, can influence the efficacy of management practices (D’Emden et al., 2008; Liu et al., 2018). For breeders and agronomists, this insight underscores the importance of multi-year trials to capture the full spectrum of genotype-environment interactions and avoid overfitting predictive models to a single growing season.

### 4.2 Environmental and temporal variability of sorghum grain quality traits

The inter-annual variability observed in the PCA analysis indicates that environmental conditions play a significant role in shaping trait expression. The distinct clustering of genotypes across 2023 and 2024 highlights the importance of considering temporal variability in breeding programs. Stable genotypes that consistently exhibit desirable traits under diverse conditions should be prioritized for further development (Faye et al., 2019). Additionally, the PCA results for 2023 and 2024 suggest that management practices influence trait variability, as indicated by the clustering of treatments. The diverse clusters suggest that specific agronomic interventions contribute to distinct trait expression patterns, highlighting the importance of management-driven trait selection and the potential of optimizing agronomic practices to enhance crop resilience (Gaudin et al., 2015; Shakoor et al., 2016; Sellami et al., 2024). Moreover, the significant differences in precipitation and temperature between the two years illustrate the need for models that are resilient to environmental fluctuations. The distinct year-to-year clustering in the PCA closely aligns with shifts in key climate variables shown in **Figure 4** and **Supplementary Figure S1**, suggesting that grain composition responds sensitively to changes in cumulative heat, rainfall distribution, and solar radiation. While our study did not directly model trait-climate correlations, the way shifts in weather conditions track and the PCA patterns indicates that climatic covariates could be quantitatively integrated in future analyses to better explain trait variation. Incorporating weather forecasts into management decisions could help mitigate the risks associated with variable growing conditions (Challinor et al., 2014).

### 4.3 UAV-derived indices as early predictors

Across DAS, both correlation screening and cross-validated models indicated measurable signal between UAV-derived RGB/MS time serie indices (e.g., NDVI, VARI/RGVI, NDCI) and some biochemical (CP, LysG, SC, CF) traits analyzed here (**Supplementary Figures S2**, **S4**, **S5**). The SHAP analyses reveal a group of indices that consistently drives prediction performance across DAS for each trait, with their relative importance differing by both trait and developmental stage (**Supplementary Figure S3**). These findings highlight the potential of UAVs as non-invasive tools for early prediction of grain quality traits. UAV-derived indices offer the advantage of high spatial resolution, allowing for plot-level monitoring and real-time decision-making during the growing season (Gano et al., 2021; Chatterjee et al., 2023; Aviles Toledo et al., 2024; Dembele et al., 2024). Previous research already showed the possible prediction of sorghum grain composition from indoor NIR and hyperspectral imagery (Huang et al., 2021; Hacisalihoglu et al., 2022). We refrain from drawing broad agronomic conclusions at this stage, as the highest predictive accuracy achieved (LysG R² = 0.43; **Supplementary Figure S2**) remains insufficient for reliable decision support.

This interpretation is reinforced by the quantitative performance losses observed when models are restricted to preharvest-only inputs (**Supplementary Figure S6**; **Supplementary Table S7**), confirming that UAV and physiological traits alone capture only a limited fraction of final grain biochemical variation. Instead, future work should focus on integrating multi-year and multi-location UAV datasets into early-stage predictive models to better inform in-season management and optimization strategies (Sun et al., 2022; Pancorbo et al., 2023; Castilho Silva et al., 2025). Including other grain quality traits as input features greatly improved prediction of [amylose/CP], supporting our cost-reduction strategy: a small panel of seed measurements can replace numerous assays while preserving model accuracy.

### 4.4 Machine learning model performance and implications

Our results support a cost-aware strategy that integrates a small, information-rich subset of grain quality traits among model inputs while estimating the remainder, thereby minimizing reliance on full laboratory panels. Examination of mean SHAP values and feature-importance rankings (**Supplementary Figures**) shows substantial predictive redundancy among traits: with crude protein (CP) and lysine measured directly in grain (LysG) as inputs with other traits including UAV and ground truth, the models accurately recover lysine predicted from protein (LysP) and starch (SC); likewise, with LysG plus LysP, we can accurately estimate crude fat (CF), protein (CP), amylose in grain (AMLG), and amylose in starch (AMLS). These patterns are consistent across spatial, temporal, and cross-validation schemes, indicating that the signal is robust to sample partitioning and likely to generalize operationally.

Moreover, a direct quantitative comparison of models trained with all features versus preharvest-only inputs (**Supplementary Figure S6**; **Supplementary Table S6**) shows that restricting models to early-season traits leads to large reductions in predictive performance for most biochemical traits, exceeding 75% for starch and amylose traits and approximately 50% for crude protein. Based on this, we propose a platform that first elicits only the essential reference assays, LysP and LysG from the chemistry lab, then uses trained models to impute the remaining five traits (CP, SC, CF, AMLS, AMLG). This approach lowers per-sample assay costs and turnaround time while preserving decision quality, and it can be coupled with periodic full-panel spot checks for recalibration and drift monitoring to maintain accuracy as environments, genetics, and management practices evolve.

The train-test split based on year (training on 2023 data, testing on 2024 data) was specifically designed to reflect real-world temporal separation and evaluate the models’ robustness under varying conditions. However, the observed performance gap suggests that relying solely on temporal train-test splits may lead to suboptimal results due to year-to-year variability in environmental conditions (Gano et al., 2021). The drop in model performance highlights the challenges of generalizing predictions across different growing seasons, which often present varying environmental and weather conditions (Zhao et al., 2013; Urban et al., 2016). To address this, 5-fold cross-validation was employed as an additional validation method to provide a broader assessment of model stability. By shuffling and splitting the data randomly across years, cross-validation helps to reduce the sensitivity to any single year’s conditions, often resulting in higher accuracy scores (Wani et al., 2018; Haile et al., 2021). However, this higher accuracy does not necessarily translate to better real-world performance, as cross-validation does not preserve temporal integrity. This overestimation can occur because the shuffled nature of cross-validation inadvertently mixes conditions from different years, which may not be available in future predictions.

Our study shows that traits such as LysG and CF can be reliably estimated when training and testing occur under mixed-year conditions, as in cross-validation and spatial validation, where environmental backgrounds are partially shared across folds. However, when the model is asked to predict trait expression in a completely new year, as in the temporal validation setting, unobserved year-specific environmental variation introduces shifts that cannot be captured from a single training season. The low temporal validation performance therefore does not indicate that these traits are fundamentally unpredictable, but rather that robust generalization across years requires either multi-year training data or modeling approaches that explicitly incorporate environmental variability. Cross-validation and spatial validation should thus be interpreted as evidence of the models’ capacity to learn trait relationships under overlapping environmental conditions, while temporal validation provides the most realistic benchmark for operational prediction across years, a scenario where greater caution and additional data are needed.

An additional limitation of this study is the relatively small sample size, which constrains model complexity and increases susceptibility to overfitting. As a result, model stability and generalizability remain provisional, underscoring the need for expanded multi-year datasets to strengthen confidence in trait predictions.

### 4.5 Practical implications for precision agriculture and breeding

The insights gained from this study provide actionable recommendations for breeders, agronomists, and farmers. By identifying optimal genotype–management combinations, stakeholders can implement targeted strategies to improve both yield and nutritional quality (Beres et al., 2020; Reynolds et al., 2021). For example, Hybrid A may be suited for protein-rich applications, while Hybrid B could be promoted in markets emphasizing lysine content. UAV-based monitoring can further reduce the cost and time of traditional field assessments, making precision agriculture more accessible (Shu et al., 2022; Wu et al., 2025). These tools not only optimize resource allocation but also support data-driven decision-making for more sustainable and climate-resilient practices. Machine-learning models could further enhance these predictions by enabling real-time adjustments based on environmental and phenotypic data, and their continued development will improve the precision and adaptability of recommendations (Sapkota et al., 2020; Gano et al., 2024), though current early prediction models still require refinement.

This study was conducted as part of an ongoing five-year sorghum trial led by our group in collaboration with the National Sorghum Producers. Within this broader effort, the present work represents an initial step toward reducing the costs of biochemical trait assessment. The models and data analyzed here, together with future datasets, will contribute to the development of software tools that enable rapid estimation of grain composition with minimal reliance on laboratory reference assays. Our objective differs from early prediction; we assume harvest has occurred and wish to reduce laboratory costs. Thus, including other grain composition traits, as predictors is intentional and practical. We prevented true data leakage by always excluding the target trait itself from the inputs. This ablation shows which traits provide redundant information and where costs can be saved without compromising accuracy.

Wet-chemistry assays for grain biochemical traits, particularly amino acid profiling are among the most expensive routine measurements in sorghum breeding pipelines. Based on typical university analytical laboratory fees, a full wet-chemistry panel can cost $150–$300 per sample, whereas directly measuring only LysG and LysP reduces analytical costs to approximately $80–$100 per sample, representing a 40–60% reduction (A Fee Schedule, 2024, The University of Missouri Agriculture Experiment Station Chemical Laboratories, n.d.). At the scale of large breeding trials, these savings become substantial, although they must be balanced against the decision-making risks associated with prediction errors, especially for traits with weaker temporal-validation performance. To mitigate these risks, we recommend periodic full-panel validation checks, the incorporation of prediction uncertainty thresholds, and limiting model-based estimates to advisory or screening roles when high precision is required. For context, rapid NIR assays costing $40–$60 per sample offer inexpensive complementary data streams but cannot replace wet-chemistry measurements needed for calibration (Welcome to Foragelab; Available online at: https://www.vtdairy.dasc.vt.edu/content/dam/vtdairy_dasc_vt_edu/documents/cow-college/2011-cc/14-stallings-paper.pdf).

Expanding the scope of prediction to include phenolic compounds, tannins, fiber fractions, minerals (e.g., Fe, Zn, P, Mg), and soluble sugars will further enhance the utility of such tools for breeding and management. Robust predictive platforms will accelerate trait evaluation, support nutritional and industrial applications, and provide breeders with cost-effective approaches for improving sorghum grain quality. Nonetheless, accurate estimation of grain composition ultimately depends on a clear understanding of both predictive models and trait interactions to ensure reliability.

## 5 Conclusion

This study demonstrates the significant potential of integrating machine learning models, ground truth, and UAV-based data for predicting key grain biochemical traits in commercial sorghum hybrids under diverse management practices. Our findings emphasize the critical role of genotype-treatment interactions, with Hybrid A and Hybrid B showing differential responses to combinations of cover cropping, precision nitrogen application, and tillage practices. The superior predictive performance of models like ElasticNet and LASSO regression underscores their ability to capture the multifactorial nature of biochemical traits, such as crude protein and amylose, particularly when key physiological, agronomic, and UAV-derived features are incorporated into the models. Moreover, the temporal variability observed between 2023 and 2024 highlights the necessity of robust, multi-year trials to ensure model generalizability and practical applicability in real-world scenarios. While partial dependence plots helped illustrate linear and nonlinear model-learned relationships, we acknowledge that PDPs assume independence among features, which may not fully hold in biological datasets; therefore, PDP patterns should be interpreted as approximations of marginal model behavior rather than isolated causal responses.

This research provides actionable insights for breeders, agronomists, and precision agriculture practitioners seeking to enhance sorghum productivity and nutritional quality. By identifying optimal genotype-management combinations and leveraging non-invasive UAV monitoring, this study offers a scalable framework for decision-making and targeted field interventions. The observed challenges in predicting year-to-year trait stability, particularly under varying environmental conditions, call for further exploration of weather-resilient predictive models. Future studies should focus on integrating long-term weather forecasts, soil health assessments, and advanced feature selection techniques to refine predictive accuracy and optimize the selection of high-performing sorghum hybrids tailored for specific environments and end-use applications​.

## Data Availability

The datasets presented in this study can be found in online repositories, on Figshare https://figshare.com/s/2765f89c7ea840e5c6be?file=59367320. The names of the repository/repositories and accessionnumber(s) can be found in the article/**Supplementary Material**.
