# Supplementary material for: Modeling grain biochemical composition traits of commercial sorghum hybrids under diverse management practices
Source: Front Plant Sci. 2026 Feb 16;17:1768456. doi: 10.3389/fpls.2026.1768456 (PMC12950713; doi:10.3389/fpls.2026.1768456)
Supplement: Supplementary file 1 [file DataSheet1.docx]

***Supplementary Material***

6. Supplementary Material


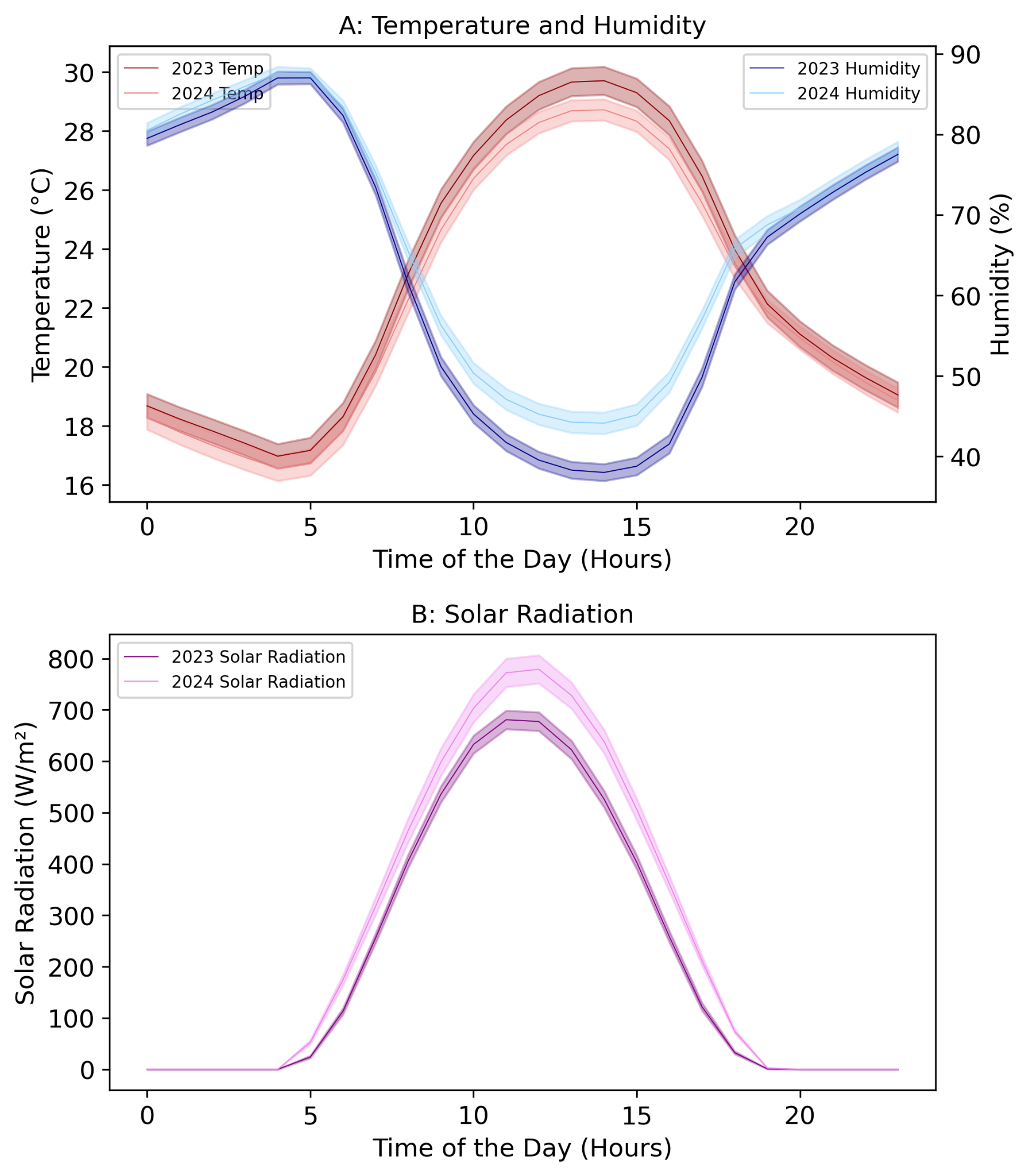


Figure S1. **Hourly Weather Patterns in The Field Experiment in Missouri: Temperature, Humidity, and Solar Radiation Trends.** The first panel (A) illustrates the fluctuations in temperature (°C) and humidity (%), while the second panel (B) presents the solar radiation (W/m²). The solid lines represent the average values, and the shaded regions indicate the standard error.

Table S1. **Herbicide and Nitrogen Quantities Applied to Promote Plant Growth Under Different Management Practices in 2023 and 2024**.

|  |  | Herbicide application rate | | | | | | | Nitrogen rate |
| --- | --- | --- | --- | --- | --- | --- | --- | --- | --- |
| Year | Management Practices | PE | PM | PA | PBII | PA4 | PB | P2,4 | NR |
| 2023 | Control | 0.2 | 0.4 | 1.7 | 0 | 0 | 0 | 0 | 224 |
|  | NT | 0.2 | 0.4 | 1.7 | 0 | 0 | 2.3 | 1.1 | 224 |
|  | PN | 0.2 | 0.4 | 1.7 | 0 | 0 | 0 | 0 | 207 |
|  | CC | 0.2 | 0.4 | 1.7 | 0 | 0 | 0 | 0 | 224 |
|  | NTPN | 0.2 | 0.4 | 1.7 | 0 | 0 | 2.3 | 1.1 | 207 |
|  | NTCC | 0.2 | 0.4 | 1.7 | 0 | 0 | 2.3 | 1.1 | 224 |
|  | CCPN | 0.2 | 0.4 | 1.7 | 0 | 0 | 0 | 0 | 207 |
|  | NTCCPN | 0.2 | 0.4 | 1.7 | 0 | 0 | 2.3 | 1.1 | 205 |
| 2024 | Control | 0 | 0 | 0 | 1.4 | 2.3 | 3 | 1.1 | 224 |
|  | NT | 0 | 0 | 0 | 1.4 | 2.3 | 3 | 1.1 | 224 |
|  | PN | 0 | 0 | 0 | 1.4 | 2.3 | 3 | 1.1 | 149 |
|  | CC | 0 | 0 | 0 | 1.4 | 2.3 | 3 | 1.1 | 224 |
|  | NTPN | 0 | 0 | 0 | 1.4 | 2.3 | 3 | 1.1 | 170 |
|  | NTCC | 0 | 0 | 0 | 1.4 | 2.3 | 3 | 1.1 | 224 |
|  | CCPN | 0 | 0 | 0 | 1.4 | 2.3 | 3 | 1.1 | 148 |
|  | NTCCPN | 0 | 0 | 0 | 1.4 | 2.3 | 3 | 1.1 | 139 |

NR : Nitrogen Rate (kg ha^-1^); PBII : Herbicide Brawl II (L ha^-1^) ; PA4 : Herbicide AAtrex4L (L ha^-1^); PB : Herbicide Buccaneer 5 Extra (L ha^-1^); P2,4: Herbicide 2,4-D (L ha^-1^); PE : Herbicide Explorer (L ha^-1^); PA : Herbicide Atrazine4L (L ha^-1^); PM : Herbicide Moxy2E (L ha^-1^).

Herbicides, specifically Buccaneer 5Extra (active ingredient: Glyphosate) and 2,4-D LV4 (active ingredient: 2-ethylhexyl ester of 2,4-Dichlorophenoxyacetic acid) were used before planting in both years to manage the weeds. In the first year, only the no-till fields were deemed necessary to spray for weed management but in 2024 all fields received pre-planting treatment. In 2023, once the sorghum was in the five-leaf stage, a combination of Explorer (active ingredient: Mesotrione), Atrazine4L (active ingredient: Atrazine), and Moxy2E (active ingredient: Octanoic acid ester of bromoxynil) were used for weed management; this had an adverse effect of heavy leaf bleaching, but after one week the sorghum returned to normal growth. The chemicals used for 2024 were changed to Brawl II (active ingredient: S-metolachlor) and AAtrex4L (active ingredient: Atrazine). We selected herbicides commonly used in sorghum and maize farming, choosing combinations that cover the widest range of weed species. Some herbicides are highly selective, for example, targeting only broad-leaf plants or grasses, so combinations were necessary to achieve broad coverage. The variety of chemicals used specialize in either pre-emergent, post-emergent, systemic, or a combination of features. In all cases, the herbicides were mixed into a liquid solution and sprayed on the fields using a tractor at the rates shown in Table 1.

Table S2. **Flight and Data Acquisition Summary.** For each mission we report date, crop growth stage, altitude above ground level (AGL), ground-sampling distance (GSD; cm/pixel), local solar time imaging window, reflectance calibration (panel/irradiance) performed before and after flights, radiometric correction, ground control points (GCPs), photogrammetry software and version, and sensors used. Abbreviations: DAS, days after sowing; GSD, ground-sampling distance; AGL, above ground level; R, red; G, green; B, blue; RE, red edge; NIR, near infrared; RGB, three-band visible camera; MS, multispectral camera; RTK, real-time kinematic; GCPs, ground control points; ODM, OpenDroneMap; Reflect. Calib., reflectance panel/irradiance calibration; Rad. Cor., radiometric correction; Flo-Mat, flowering to maturity. When two GSD values are listed (e.g., 2.18 & 3.6), they correspond to RGB and MS mosaics, respectively. Times are local (09:00–13:00). Approximate altitudes are noted as “~50 m AGL.”

| Flight # | Date | DAS | Growth | Altitude | GSD(cm/pixel) | Time | Reflect. Calib. | Rad. Cor. | GCPs | Software | Sensors |
| --- | --- | --- | --- | --- | --- | --- | --- | --- | --- | --- | --- |
| 1 | 6/9/23 | 0 | Bare soil | ~50 m AGL | 2.18 & 3.6 | 9AM-1PM | bef. & aft. Flight | Yes | RTK targets | ODM 2.9.1 | RGB & MS |
| 2 | 6/15/23 | 0 | Bare soil | ~50 m AGL | 2.18 & 3.6 | 9AM-1PM | bef. & aft. Flight | Yes | RTK targets | ODM 2.9.1 | RGB & MS |
| 3 | 6/21/23 | 5 | Emergence | ~50 m AGL | 2.18 & 3.6 | 9AM-1PM | bef. & aft. Flight | Yes | RTK targets | ODM 2.9.1 | RGB & MS |
| 4 | 6/29/23 | 13 | Seedling | ~50 m AGL | 2.18 & 3.6 | 9AM-1PM | bef. & aft. Flight | Yes | RTK targets | ODM 2.9.1 | RGB & MS |
| 5 | 7/7/23 | 21 | Vegetative | ~50 m AGL | 2.18 & 3.6 | 9AM-1PM | bef. & aft. Flight | Yes | RTK targets | ODM 2.9.1 | RGB & MS |
| 6 | 7/12/23 | 26 | Vegetative | ~50 m AGL | 2.18 & 3.6 | 9AM-1PM | bef. & aft. Flight | Yes | RTK targets | ODM 2.9.1 | RGB & MS |
| 7 | 7/17/23 | 31 | Vegetative | ~50 m AGL | 2.18 & 3.6 | 9AM-1PM | bef. & aft. Flight | Yes | RTK targets | ODM 2.9.1 | RGB & MS |
| 8 | 7/27/23 | 41 | Booting | ~50 m AGL | 2.18 & 3.6 | 9AM-1PM | bef. & aft. Flight | Yes | RTK targets | ODM 2.9.1 | RGB & MS |
| 9 | 8/4/23 | 49 | Booting | ~50 m AGL | 2.18 & 3.6 | 9AM-1PM | bef. & aft. Flight | Yes | RTK targets | ODM 2.9.1 | RGB & MS |
| 10 | 8/10/23 | 55 | Flowering | ~50 m AGL | 2.18 & 3.6 | 9AM-1PM | bef. & aft. Flight | Yes | RTK targets | ODM 2.9.1 | RGB & MS |
| 11 | 8/17/23 | 62 | Flowering | ~50 m AGL | 2.18 & 3.6 | 9AM-1PM | bef. & aft. Flight | Yes | RTK targets | ODM 2.9.1 | RGB & MS |
| 12 | 8/22/23 | 67 | Flo-Mat | ~50 m AGL | 2.18 & 3.6 | 9AM-1PM | bef. & aft. Flight | Yes | RTK targets | ODM 2.9.1 | RGB & MS |
| 13 | 8/31/23 | 76 | Flo-Mat | ~50 m AGL | 2.18 & 3.6 | 9AM-1PM | bef. & aft. Flight | Yes | RTK targets | ODM 2.9.1 | RGB & MS |
| 14 | 7/2/24 | 26 | Vegetative | ~50 m AGL | 1.45 | 9AM-1PM | bef. & aft. Flight | Yes | RTK targets | ODM 2.9.1 | RGB |
| 15 | 8/1/24 | 56 | Flowering | ~50 m AGL | 1.45 | 9AM-1PM | bef. & aft. Flight | Yes | RTK targets | ODM 2.9.1 | RGB |
| 16 | 9/4/24 | 90 | Maturity | ~50 m AGL | 1.45 | 9AM-1PM | bef. & aft. Flight | Yes | RTK targets | ODM 2.9.1 | RGB |
| 17 | 6/21/24 | 15 | Vegetative | ~50 m AGL | 2.29 | 9AM-1PM | bef. & aft. Flight | Yes | RTK targets | ODM 2.9.1 | MS |
| 18 | 7/10/24 | 34 | Vegetative | ~50 m AGL | 2.29 | 9AM-1PM | bef. & aft. Flight | Yes | RTK targets | ODM 2.9.1 | MS |
| 19 | 7/19/24 | 43 | Booting | ~50 m AGL | 2.29 | 9AM-1PM | bef. & aft. Flight | Yes | RTK targets | ODM 2.9.1 | MS |
| 20 | 8/5/24 | 60 | Flowering | ~50 m AGL | 2.29 | 9AM-1PM | bef. & aft. Flight | Yes | RTK targets | ODM 2.9.1 | MS |
| 21 | 8/19/24 | 74 | Flowering | ~50 m AGL | 2.29 | 9AM-1PM | bef. & aft. Flight | Yes | RTK targets | ODM 2.9.1 | MS |
| 22 | 8/30/24 | 85 | Maturity | ~50 m AGL | 2.29 | 9AM-1PM | bef. & aft. Flight | Yes | RTK targets | ODM 2.9.1 | MS |
| 23 | 9/12/24 | 98 | Maturity | ~50 m AGL | 2.29 | 9AM-1PM | bef. & aft. Flight | Yes | RTK targets | ODM 2.9.1 | MS |
| 24 | 9/25/24 | 111 | Maturity | ~50 m AGL | 2.29 | 9AM-1PM | bef. & aft. Flight | Yes | RTK targets | ODM 2.9.1 | MS |

Table S3. **Key vegetation indices used in the study and derived from UAV RGB sensor data.** Abr. : abbreviation, Ref.: references, B, G, R: reflectance at blue, green, and red bands respectively

| Plant indices | Abb. | Formulas | Ref. |
| --- | --- | --- | --- |
| Color Index of Vegetation | CIVE | (0.441 × R) − (0.881 × G) + (0.385 × B) + 18.78745 | (Kataoka et al., 2004) |
| Visible Atmospherically Resistant Index | VARI | (G − R​) / (G + R − B) | (Gitelson et al., 2003) |
| Redness Index | RI | R^2^​ / (B × G)^3^ | (rgb_indices function - RDocumentation, n.d.) |
| Brightness Index | BI | sqrt ((R^2^​+G^2^+B^2^) / 3 | (rgb_indices function - RDocumentation, n.d.) |
| Visible Vegetation index | VVI | (1− (R − 30) / (R + 30)) × (1− (G − 50) / (G + 50)) × (1− (B − 1) / (B + 1)) | (Reudenbach, 2019) |
| Red Chromatic Coordinate Index | RCC | R / (R + G +B) | (De Swaef et al., 2021) |
| Green Leaf Index | GLI | (2 × G − R − B) / (2 × G + R + B) | (Louhaichi et al., 2001) |
| Shape Index | SHP | 2 × (R − G − B) / (G − B) | (rgb_indices function - RDocumentation, n.d.) |
| Overall Hue Index | HUE | atan (2 × (R − G − B) / 30.5 × (G − B)) | (rgb_indices function - RDocumentation, n.d.) |

Table S4. Key vegetation indices used in the study and derived from UAV MS sensors data.

| Plant Index | Abb. | Formula (using band means) | Notes / Constants | Ref. |
| --- | --- | --- | --- | --- |
| Normalized Difference Vegetation Index | NDVI | (NIR − R) / (NIR + R) |  | (Grbović et al., 2025) |
| Green NDVI | gNDVI | (NIR − G) / (NIR + G) |  | (Grbović et al., 2025) |
| Ratio Vegetation Index | RVI | NIR / R |  | (List of available Indices, n.d.) |
| Green Chlorophyll Index | GCI | (NIR / G) − 1 |  | (List of available Indices, n.d.) |
| Red–Green Vegetation Index | RGVI | R / G |  | (List of available Indices, n.d.) |
| Difference Vegetation Index | DVI | NIR − R |  | (List of available Indices, n.d.) |
| Soil Adjusted Vegetation Index | SAVI | ((NIR − R) / (NIR + R + L)) × (1 + L) | L = 0.5 | (Grbović et al., 2025) |
| Modified SAVI | MSAVI | 0.5 × ((2 × NIR + 1) − √((2 × NIR + 1)² − 8 × (NIR − R))) |  | (Alphabetical List of Spectral Indices, n.d.) |
| Optimized SAVI | OSAVI | (NIR − R) / (NIR + R + 0.16) |  | (Alphabetical List of Spectral Indices, n.d.) |
| Renormalized DVI | RDVI | √((NIR − R)² / (NIR + R)) |  | (Alphabetical List of Spectral Indices, n.d.) |
| Transformed VI | TVI | 60 × (NIR − G) − 100 × (R − G) |  | (Alphabetical List of Spectral Indices, n.d.) |
| Transformed SAVI | TSAVI | (a × (NIR − a × R − b)) / (a × NIR + R − a × b) | a = 0.96916, b = 0.084726 | (Alphabetical List of Spectral Indices, n.d.) |
| Perpendicular VI | PVI | (NIR − a × R − b) / √(1 + a²) | a = 0.96916, b = 0.084726 | (Alphabetical List of Spectral Indices, n.d.) |
| Adjusted TSAVI | ATSAVI | (a × (−a × R − b)) / (a × NIR + R − a × b + x × (1 + a²)) | a = 0.96916, b = 0.084726, x = 0.08 | (Alphabetical List of Spectral Indices, n.d.) |
| Normalized Difference Water Index | NDWI | (G − NIR) / (G + NIR) |  | (Alphabetical List of Spectral Indices, n.d.) |
| Normalized Pigment Chlorophyll Index | NPCI | (R − B) / (R + B) |  | (Alphabetical List of Spectral Indices, n.d.) |
| Simple Ratio Pigment Index | SRPI | B / R |  | (Alphabetical List of Spectral Indices, n.d.) |
| Green Ratio VI | RVI₂ | NIR / G |  | (Alphabetical List of Spectral Indices, n.d.) |
| Modified Chlorophyll Absorption Ratio Index | MCARI | (RE − R − 0.2 × (RE − G)) × (RE / R) |  | (Shammi et al., 2024) |
| MCARI_1 | MCARI₁ | 1.2 × (2.5 × (NIR − R) − 1.3 × (NIR − G)) |  | (Shammi et al., 2024) |
| MCARI_2 | MCARI₂ | 1.5 × (2.5 × (NIR − R) − 1.3 × (NIR − G)) × (2 × NIR + 1)² − (6 × NIR − 5 × R) − 0.5 |  | (Shammi et al., 2024) |
| Modified TVI-1 | MTVI₁ | 1.2 × (1.2 × (NIR − G) − 2.5 × (R − G)) |  | (Alphabetical List of Spectral Indices, n.d.) |
| Modified TVI-2 | MTVI₂ | 1.5 × (1.2 × (NIR − G) − 2.5 × (R − G)) × (2 × NIR + 1)² − (6 × NIR − 5 × R) − 0.5 |  | (Alphabetical List of Spectral Indices, n.d.) |
| MCARI/MTVI₂ | R-MCARI/MTVI₂ | MCARI / MTVI₂ |  | (Shammi et al., 2024) |
| Enhanced VI | EVI | (NIR − R) / (NIR + 6 × R − 7.5 × B + 1) |  | (Grbović et al., 2025) |
| DATT Index | DATT | (NIR − RE) / (NIR − R) |  | (List of available Indices, n.d.) |
| Normalized Difference Chlorophyll Index | NDCI | (RE − G) / (RE + G) |  | (Shammi et al., 2024) |
| Plant Senescence Reflectance Index | PSRI | (R − G) / RE |  | (List of available Indices, n.d.) |
| Structure-Insensitive Pigment Index | SIPI | (NIR − B) / (NIR + R) |  | (List of available Indices, n.d.) |
| Shadow-Adjusted PV Index | SPVI | 0.4 × 3.7 × (NIR − R) − 1.2 × (G − R) |  | (List of available Indices, n.d.) |
| Transformed Chlorophyll Absorption Ratio Index | TCARI | 3 × ((RE − R) − 0.2 × (RE − G) × (RE / R)) |  | (Shammi et al., 2024) |
| TCARI/OSAVI ratio | R-TCARI/OSAVI | TCARI / OSAVI |  | (Shammi et al., 2024) |
| Red-Edge Ratio Index | RERI | (RE − R) / NIR |  | (List of available Indices, n.d.) |
| Red-Edge NDVI | NDRE | (NIR − RE) / (NIR + RE) |  | (Shammi et al., 2024) |
| MERIS Terrestrial Chlorophyll Index | MTCI | (NIR − RE) / (RE − R) |  | (List of available Indices, n.d.) |
| Red-Edge Chlorophyll Index | RECI | (NIR / RE) − 1 |  | (List of available Indices, n.d.) |
| Normalized Excess Green | NExG | (2 × G − R − B) / (G + R + B) |  | (Memon et al., 2025) |
| Normalized Green–Red Difference | NGRDI | (G − R) / (G + R) |  | (Memon et al., 2025) |
| Enhanced NDVI | ENDVI | (NIR + G − 2 × B) / (NIR + G + 2 × B) |  | (Radócz et al., 2023) |
| Anthocyanin Reflectance Index 2 | ARI₂ | NIR × (1 / G − 1 / RE) |  | (Alphabetical List of Spectral Indices, n.d.) |
| Carotenoid Reflectance Index 2 | CRI₂ | (1 / G) − (1 / RE) |  | (Alphabetical List of Spectral Indices, n.d.) |
| Normalized RVI | NRVI | (1 / RVI − 1) / (1 / RVI + 1), RVI = NIR / R |  | (Alphabetical List of Spectral Indices, n.d.) |
| Optimized SAVI (scaled) | OSAVI₂ | ((1 + 0.16) × (NIR − R)) / (NIR + R + 0.16) |  | (Alphabetical List of Spectral Indices, n.d.) |
| Enhanced VI (variant) | EVI₃ | (2.5 × (NIR − R)) / (NIR + 6 × R − 7.5 × B + 1) |  | (Alphabetical List of Spectral Indices, n.d.) |
| Modified EVI | MEVI | (2.5 × (NIR − R)) / (NIR + 2.4 × R + 1) |  | (Alphabetical List of Spectral Indices, n.d.) |
| VARI (NIR-based) | VARI_NIR | (NIR − G) / (NIR + G − B) |  | (Alphabetical List of Spectral Indices, n.d.) |
| Transformed VI (sqrt form) | TVI₂ | √(NDVI + 0.5) |  | (Alphabetical List of Spectral Indices, n.d.) |
| MCARI/OSAVI | MCARI/OSAVI | MCARI / OSAVI |  | (Alphabetical List of Spectral Indices, n.d.) |
| TCARI/OSAVI | TCARI/OSAVI | TCARI / OSAVI |  | (Alphabetical List of Spectral Indices, n.d.) |
| Wide Dynamic Range VI | WDRVI | (L × NIR − R) / (L × NIR + R) | L = 0.12 | (Alphabetical List of Spectral Indices, n.d.) |

Table S5. **Heteroscedasticity-Robust ANOVA (Welch) Results for Genotype, Treatment, and Year Effects Across Traits.**

Residuals were approximately normal (Shapiro–Wilk: CP p = 0.9287; AMLS p = 0.7506), but Levene’s test indicated heteroscedasticity across groups (p < 2.2×10^-16^ across traits). Accordingly, we based all inferences on Welch’s ANOVA, which relaxes the equal-variance assumption via the Welch–Satterthwaite correction and is appropriate under unequal variances and sample sizes. This approach directly addresses trait-specific variability and preserves valid false positive error control under variance heterogeneity.

|  |  |  |  |  | Shap. Wilk | Levene Test |
| --- | --- | --- | --- | --- | --- | --- |
| Response |  | Df | F | Pr(>F) | p-value | Pr(>F) |
| CP | (Intercept) | 1 | 2.97E+05 | 2.2E-16*** | 0.9287 | < 2.2e-16 *** |
| CP | Genotype (G) | 1 | 2.87E+00 | 1.00E-01 |  |  |
| CP | Treatment (T) | 7 | 9.42E+00 | 2.69E-6*** |  |  |
| CP | Year (Y) | 1 | 2.20E+02 | 6.73E-16*** |  |  |
| CP | G * T | 7 | 7.12E-01 | 6.62E-01 |  |  |
| CP | G * Y | 1 | 1.74E-01 | 6.80E-01 |  |  |
| CP | T * Y | 7 | 1.36E+01 | 5.391E-8*** |  |  |
| CP | G * T * Y | 7 | 4.23E-01 | 8.81E-01 |  |  |
| CP | Residuals | 32 |  |  |  |  |
| AMLS | (Intercept) | 1 | 433.4105 | < 2.2e-16 *** | 0.7506 | < 2.2e-16 *** |
| AMLS | Genotype (G) | 1 | 0.0387 | 0.8453026 |  |  |
| AMLS | Treatment (T) | 7 | 5.181 | 0.0005077 *** |  |  |
| AMLS | Year (Y) | 1 | 0.5463 | 0.4652247 |  |  |
| AMLS | G * T | 7 | 6.2723 | 0.0001125 *** |  |  |
| AMLS | G * Y | 1 | 0.0063 | 0.9374284 |  |  |
| AMLS | T * Y | 7 | 0.5119 | 0.8186839 |  |  |
| AMLS | G * T * Y | 7 | 0.1792 | 0.9877837 |  |  |
| AMLS | Residuals | 32 |  |  |  |  |
| AMLG | (Intercept) | 1 | 524.4459 | < 2.2e-16 *** | 0.994 | < 2.2e-16 *** |
| AMLG | Genotype (G) | 1 | 0.0529 | 0.819474 |  |  |
| AMLG | Treatment (T) | 7 | 4.1188 | 0.002499 ** |  |  |
| AMLG | Year (Y) | 1 | 0.2193 | 0.642744 |  |  |
| AMLG | G * T | 7 | 7.6607 | 1.957e-05 *** |  |  |
| AMLG | G * Y | 1 | 0.0044 | 0.947265 |  |  |
| AMLG | T * Y | 7 | 0.859 | 0.54856 |  |  |
| AMLG | G * T * Y | 7 | 0.1837 | 0.986862 |  |  |
| AMLG | Residuals | 32 |  |  |  |  |
| LysP | (Intercept) | 1 | 6203.8196 | < 2.2e-16 *** | 0.2805 | < 2.2e-16 *** |
| LysP | Genotype (G) | 1 | 2.7463 | 0.107254 |  |  |
| LysP | Treatment (T) | 7 | 3.3175 | 0.009066 ** |  |  |
| LysP | Year (Y) | 1 | 70.0122 | 1.466e-09 *** |  |  |
| LysP | G * T | 7 | 3.6452 | 0.005305 ** |  |  |
| LysP | G * Y | 1 | 0.0333 | 0.85627 |  |  |
| LysP | T * Y | 7 | 18.7479 | 1.140e-09 *** |  |  |
| LysP | G * T * Y | 7 | 1.5617 | 0.182696 |  |  |
| LysP | Residuals | 32 |  |  |  |  |
| LysG | (Intercept) | 1 | 4735.9401 | < 2.2e-16 *** | 0.2921 | < 2.2e-16 *** |
| LysG | Genotype (G) | 1 | 0.1176 | 0.73393 |  |  |
| LysG | Treatment (T) | 7 | 49.9924 | 1.914e-15 *** |  |  |
| LysG | Year (Y) | 1 | 0.0001 | 0.9933 |  |  |
| LysG | G * T | 7 | 0.7857 | 0.60425 |  |  |
| LysG | G * Y | 1 | 0.03 | 0.86364 |  |  |
| LysG | T * Y | 7 | 2.9482 | 0.01681 * |  |  |
| LysG | G * T * Y | 7 | 0.4387 | 0.87035 |  |  |
| LysG | Residuals | 32 |  |  |  |  |
| SC | (Intercept) | 1 | 52313.5595 | < 2.2e-16 *** | 0.7904 | < 2.2e-16 *** |
| SC | Genotype (G) | 1 | 0.0045 | 0.9469 |  |  |
| SC | Treatment (T) | 7 | 116.2933 | < 2.2e-16 *** |  |  |
| SC | Year (Y) | 1 | 4.3368 | 0.045373 * |  |  |
| SC | G * T | 7 | 1.2243 | 0.318502 |  |  |
| SC | G * Y | 1 | 0.0121 | 0.913122 |  |  |
| SC | T * Y | 7 | 3.5552 | 0.006139 ** |  |  |
| SC | G * T * Y | 7 | 0.4554 | 0.859058 |  |  |
| SC | Residuals | 32 |  |  |  |  |
| CF | (Intercept) | 1 | 6727.2642 | < 2.2e-16 *** | 0.8982 | < 2.2e-16 *** |
| CF | Genotype (G) | 1 | 2.2513 | 0.143303 |  |  |
| CF | Treatment (T) | 7 | 2.8539 | 0.019728 * |  |  |
| CF | Year (Y) | 1 | 8.2351 | 0.007223 ** |  |  |
| CF | G * T | 7 | 0.8478 | 0.556899 |  |  |
| CF | G * Y | 1 | 0.7248 | 0.400885 |  |  |
| CF | T * Y | 7 | 0.994 | 0.453322 |  |  |
| CF | G * T * Y | 7 | 1.2725 | 0.294884 |  |  |
| CF | Residuals | 32 |  |  |  |  |

Table S6. **Post-hoc (Games–Howell) pairwise tests for genotype differences across management practices and years.** mean_A and mean_B = group means for each hybrid (Hyb) genotype A and B; diff = mean difference (mean_A − mean_B); t = Games–Howell test statistic; df = adjusted degrees of freedom; pval = p-value; sig = significance label (ns = not significant, p < 0.05 = *, p < 0.01 = **, p < 0.001 = ***).

| **trait** | **Mgmt** | **Year** | **A** | **B** | **mean_A** | **mean_B** | **diff** | **t** | **df** | **pval** | **sig** |
| --- | --- | --- | --- | --- | --- | --- | --- | --- | --- | --- | --- |
| CP | Control | 2023 | HybA | HybB | 11.58 | 11.56 | 0.02 | 0.04 | 1.03 | 0.98 | ns |
| CP | Control | 2024 | HybA | HybB | 8.78 | 10.22 | -1.44 | -1.90 | 1.67 | 0.22 | ns |
| CP | NT | 2023 | HybA | HybB | 10.93 | 9.50 | 1.43 | 7.65 | 1.24 | 0.05 | ns |
| CP | NT | 2024 | HybA | HybB | 8.62 | 8.03 | 0.59 | 0.98 | 1.59 | 0.45 | ns |
| CP | PN | 2023 | HybA | HybB | 11.29 | 10.69 | 0.59 | 1.51 | 1.45 | 0.31 | ns |
| CP | PN | 2024 | HybA | HybB | 7.75 | 8.35 | -0.60 | -1.84 | 1.02 | 0.31 | ns |
| CP | CC | 2023 | HybA | HybB | 11.40 | 10.26 | 1.14 | 2.40 | 1.00 | 0.25 | ns |
| CP | CC | 2024 | HybA | HybB | 8.51 | 7.84 | 0.66 | 1.02 | 1.09 | 0.48 | ns |
| CP | NTPN | 2023 | HybA | HybB | 11.23 | 9.81 | 1.42 | 6.77 | 1.01 | 0.09 | ns |
| CP | NTPN | 2024 | HybA | HybB | 8.71 | 7.57 | 1.14 | 1.05 | 1.45 | 0.44 | ns |
| CP | NTCC | 2023 | HybA | HybB | 10.70 | 10.59 | 0.11 | 0.13 | 1.07 | 0.92 | ns |
| CP | NTCC | 2024 | HybA | HybB | 9.26 | 9.02 | 0.24 | 0.31 | 1.99 | 0.79 | ns |
| CP | CCPN | 2023 | HybA | HybB | 11.23 | 10.03 | 1.20 | 26.40 | 1.97 | 0.00 | ** |
| CP | CCPN | 2024 | HybA | HybB | 10.56 | 9.60 | 0.96 | 5.90 | 1.44 | 0.06 | ns |
| CP | NTCCPN | 2023 | HybA | HybB | 10.57 | 9.37 | 1.21 | 4.06 | 1.40 | 0.10 | ns |
| CP | NTCCPN | 2024 | HybA | HybB | 9.80 | 7.67 | 2.13 | 1.42 | 1.84 | 0.30 | ns |
| LysG | Control | 2023 | HybA | HybB | 0.26 | 0.27 | -0.01 | -0.69 | 1.03 | 0.61 | ns |
| LysG | Control | 2024 | HybA | HybB | 0.26 | 0.28 | -0.02 | -1.14 | 1.33 | 0.42 | ns |
| LysG | NT | 2023 | HybA | HybB | 0.25 | 0.24 | 0.00 | 1.65 | 1.36 | 0.29 | ns |
| LysG | NT | 2024 | HybA | HybB | 0.26 | 0.25 | 0.01 | 2.97 | 1.49 | 0.14 | ns |
| LysG | PN | 2023 | HybA | HybB | 0.26 | 0.26 | 0.00 | -0.05 | 1.22 | 0.97 | ns |
| LysG | PN | 2024 | HybA | HybB | 0.25 | 0.26 | -0.01 | -2.67 | 1.01 | 0.23 | ns |
| LysG | CC | 2023 | HybA | HybB | 0.26 | 0.26 | 0.00 | 0.48 | 1.61 | 0.69 | ns |
| LysG | CC | 2024 | HybA | HybB | 0.26 | 0.25 | 0.01 | 0.35 | 1.89 | 0.76 | ns |
| LysG | NTPN | 2023 | HybA | HybB | 0.26 | 0.25 | 0.01 | 3.30 | 1.62 | 0.11 | ns |
| LysG | NTPN | 2024 | HybA | HybB | 0.27 | 0.26 | 0.01 | 0.68 | 1.39 | 0.59 | ns |
| LysG | NTCC | 2023 | HybA | HybB | 0.25 | 0.25 | 0.00 | 0.81 | 1.00 | 0.57 | ns |
| LysG | NTCC | 2024 | HybA | HybB | 0.27 | 0.27 | 0.00 | -0.18 | 1.74 | 0.88 | ns |
| LysG | CCPN | 2023 | HybA | HybB | 0.26 | 0.25 | 0.01 | 9.13 | 1.06 | 0.06 | ns |
| LysG | CCPN | 2024 | HybA | HybB | 0.28 | 0.28 | 0.00 | -0.46 | 1.28 | 0.71 | ns |
| LysG | NTCCPN | 2023 | HybA | HybB | 0.24 | 0.24 | 0.00 | 1.65 | 1.70 | 0.26 | ns |
| LysG | NTCCPN | 2024 | HybA | HybB | 0.27 | 0.25 | 0.02 | 1.27 | 1.25 | 0.39 | ns |
| LysP | Control | 2023 | HybA | HybB | 2.24 | 2.30 | -0.07 | -1.69 | 1.04 | 0.33 | ns |
| LysP | Control | 2024 | HybA | HybB | 2.93 | 2.72 | 0.22 | 4.33 | 1.83 | 0.06 | ns |
| LysP | NT | 2023 | HybA | HybB | 2.27 | 2.56 | -0.29 | -19.71 | 1.30 | 0.01 | * |
| LysP | NT | 2024 | HybA | HybB | 3.01 | 3.14 | -0.13 | -0.65 | 1.92 | 0.59 | ns |
| LysP | PN | 2023 | HybA | HybB | 2.28 | 2.41 | -0.13 | -3.54 | 1.70 | 0.09 | ns |
| LysP | PN | 2024 | HybA | HybB | 3.20 | 3.11 | 0.08 | 0.63 | 1.23 | 0.62 | ns |
| LysP | CC | 2023 | HybA | HybB | 2.26 | 2.50 | -0.24 | -2.34 | 1.08 | 0.24 | ns |
| LysP | CC | 2024 | HybA | HybB | 3.03 | 3.23 | -0.20 | -1.59 | 1.58 | 0.28 | ns |
| LysP | NTPN | 2023 | HybA | HybB | 2.30 | 2.51 | -0.21 | -7.19 | 1.96 | 0.02 | * |
| LysP | NTPN | 2024 | HybA | HybB | 3.07 | 3.39 | -0.31 | -1.36 | 1.80 | 0.32 | ns |
| LysP | NTCC | 2023 | HybA | HybB | 2.36 | 2.37 | -0.01 | -0.06 | 1.10 | 0.96 | ns |
| LysP | NTCC | 2024 | HybA | HybB | 2.93 | 3.03 | -0.10 | -0.78 | 1.64 | 0.53 | ns |
| LysP | CCPN | 2023 | HybA | HybB | 2.28 | 2.48 | -0.20 | -25.35 | 1.00 | 0.03 | * |
| LysP | CCPN | 2024 | HybA | HybB | 2.63 | 2.94 | -0.31 | -4.95 | 1.04 | 0.12 | ns |
| LysP | NTCCPN | 2023 | HybA | HybB | 2.31 | 2.56 | -0.26 | -4.66 | 1.16 | 0.11 | ns |
| LysP | NTCCPN | 2024 | HybA | HybB | 2.78 | 3.26 | -0.48 | -1.43 | 1.74 | 0.31 | ns |
| SC | Control | 2023 | HybA | HybB | 72.22 | 72.47 | -0.25 | -0.47 | 1.00 | 0.72 | ns |
| SC | Control | 2024 | HybA | HybB | 74.53 | 72.62 | 1.90 | 1.95 | 1.03 | 0.30 | ns |
| SC | NT | 2023 | HybA | HybB | 73.36 | 74.30 | -0.95 | -6.53 | 1.82 | 0.03 | * |
| SC | NT | 2024 | HybA | HybB | 74.46 | 74.88 | -0.42 | -0.66 | 1.15 | 0.61 | ns |
| SC | PN | 2023 | HybA | HybB | 73.14 | 72.28 | 0.86 | 1.03 | 1.01 | 0.49 | ns |
| SC | PN | 2024 | HybA | HybB | 75.41 | 74.72 | 0.70 | 1.20 | 1.16 | 0.42 | ns |
| SC | CC | 2023 | HybA | HybB | 72.68 | 72.72 | -0.03 | -0.09 | 1.88 | 0.93 | ns |
| SC | CC | 2024 | HybA | HybB | 74.59 | 74.83 | -0.24 | -0.19 | 1.56 | 0.87 | ns |
| SC | NTPN | 2023 | HybA | HybB | 72.41 | 73.84 | -1.44 | -2.66 | 1.83 | 0.13 | ns |
| SC | NTPN | 2024 | HybA | HybB | 74.82 | 74.51 | 0.31 | 0.33 | 1.35 | 0.79 | ns |
| SC | NTCC | 2023 | HybA | HybB | 73.20 | 73.00 | 0.20 | 0.38 | 1.36 | 0.76 | ns |
| SC | NTCC | 2024 | HybA | HybB | 73.61 | 73.44 | 0.17 | 0.30 | 1.19 | 0.81 | ns |
| SC | CCPN | 2023 | HybA | HybB | 72.38 | 73.25 | -0.87 | -4.97 | 1.06 | 0.12 | ns |
| SC | CCPN | 2024 | HybA | HybB | 72.74 | 72.79 | -0.04 | -0.06 | 1.79 | 0.96 | ns |
| SC | NTCCPN | 2023 | HybA | HybB | 73.68 | 74.68 | -1.00 | -7.68 | 1.13 | 0.07 | ns |
| SC | NTCCPN | 2024 | HybA | HybB | 73.41 | 75.35 | -1.95 | -1.67 | 2.00 | 0.24 | ns |
| AMLG | Control | 2023 | HybA | HybB | 21.07 | 18.80 | 2.27 | 0.93 | 1.21 | 0.50 | ns |
| AMLG | Control | 2024 | HybA | HybB | 22.49 | 20.57 | 1.91 | 1.43 | 1.10 | 0.37 | ns |
| AMLG | NT | 2023 | HybA | HybB | 21.15 | 20.80 | 0.35 | 0.38 | 1.95 | 0.74 | ns |
| AMLG | NT | 2024 | HybA | HybB | 22.09 | 22.16 | -0.07 | -0.06 | 1.98 | 0.96 | ns |
| AMLG | PN | 2023 | HybA | HybB | 20.59 | 22.70 | -2.11 | -15.98 | 1.21 | 0.02 | * |
| AMLG | PN | 2024 | HybA | HybB | 20.02 | 20.86 | -0.84 | -0.90 | 1.98 | 0.46 | ns |
| AMLG | CC | 2023 | HybA | HybB | 21.79 | 22.06 | -0.28 | -0.33 | 1.89 | 0.78 | ns |
| AMLG | CC | 2024 | HybA | HybB | 21.14 | 21.30 | -0.16 | -0.18 | 1.85 | 0.87 | ns |
| AMLG | NTPN | 2023 | HybA | HybB | 22.20 | 21.56 | 0.64 | 1.52 | 1.46 | 0.31 | ns |
| AMLG | NTPN | 2024 | HybA | HybB | 23.33 | 21.01 | 2.33 | 1.35 | 1.94 | 0.31 | ns |
| AMLG | NTCC | 2023 | HybA | HybB | 20.72 | 20.46 | 0.26 | 0.78 | 1.21 | 0.56 | ns |
| AMLG | NTCC | 2024 | HybA | HybB | 22.56 | 20.98 | 1.59 | 0.75 | 1.80 | 0.54 | ns |
| AMLG | CCPN | 2023 | HybA | HybB | 21.45 | 22.36 | -0.90 | -13.60 | 1.08 | 0.04 | * |
| AMLG | CCPN | 2024 | HybA | HybB | 20.77 | 19.66 | 1.11 | 0.89 | 1.07 | 0.53 | ns |
| AMLG | NTCCPN | 2023 | HybA | HybB | 21.07 | 21.58 | -0.51 | -0.60 | 1.28 | 0.64 | ns |
| AMLG | NTCCPN | 2024 | HybA | HybB | 21.43 | 20.65 | 0.78 | 0.54 | 1.33 | 0.66 | ns |
| AMLS | Control | 2023 | HybA | HybB | 29.17 | 25.92 | 3.26 | 1.02 | 1.24 | 0.47 | ns |
| AMLS | Control | 2024 | HybA | HybB | 30.19 | 28.33 | 1.86 | 0.97 | 1.38 | 0.47 | ns |
| AMLS | NT | 2023 | HybA | HybB | 28.82 | 27.99 | 0.83 | 0.68 | 1.99 | 0.57 | ns |
| AMLS | NT | 2024 | HybA | HybB | 29.68 | 29.60 | 0.08 | 0.05 | 1.91 | 0.97 | ns |
| AMLS | PN | 2023 | HybA | HybB | 28.15 | 31.41 | -3.26 | -8.94 | 1.72 | 0.02 | * |
| AMLS | PN | 2024 | HybA | HybB | 26.55 | 27.92 | -1.38 | -0.96 | 1.99 | 0.44 | ns |
| AMLS | CC | 2023 | HybA | HybB | 29.98 | 30.35 | -0.37 | -0.28 | 1.93 | 0.81 | ns |
| AMLS | CC | 2024 | HybA | HybB | 28.35 | 28.48 | -0.13 | -0.08 | 2.00 | 0.94 | ns |
| AMLS | NTPN | 2023 | HybA | HybB | 30.66 | 29.20 | 1.46 | 3.74 | 1.08 | 0.15 | ns |
| AMLS | NTPN | 2024 | HybA | HybB | 31.20 | 28.20 | 3.00 | 1.13 | 2.00 | 0.38 | ns |
| AMLS | NTCC | 2023 | HybA | HybB | 28.31 | 28.03 | 0.28 | 0.54 | 1.02 | 0.68 | ns |
| AMLS | NTCC | 2024 | HybA | HybB | 30.65 | 28.55 | 2.09 | 0.77 | 1.70 | 0.53 | ns |
| AMLS | CCPN | 2023 | HybA | HybB | 29.64 | 30.52 | -0.88 | -48.86 | 1.25 | 0.01 | ** |
| AMLS | CCPN | 2024 | HybA | HybB | 28.56 | 27.00 | 1.56 | 1.01 | 1.22 | 0.47 | ns |
| AMLS | NTCCPN | 2023 | HybA | HybB | 28.59 | 28.90 | -0.31 | -0.26 | 1.33 | 0.83 | ns |
| AMLS | NTCCPN | 2024 | HybA | HybB | 29.21 | 27.39 | 1.82 | 1.00 | 1.84 | 0.43 | ns |
| CF | Control | 2023 | HybA | HybB | 2.59 | 2.91 | -0.32 | -1.84 | 1.10 | 0.30 | ns |
| CF | Control | 2024 | HybA | HybB | 4.09 | 4.05 | 0.04 | 0.22 | 1.00 | 0.87 | ns |
| CF | NT | 2023 | HybA | HybB | 2.50 | 3.11 | -0.61 | -7.06 | 1.07 | 0.08 | ns |
| CF | NT | 2024 | HybA | HybB | 4.18 | 4.38 | -0.20 | -0.99 | 1.35 | 0.47 | ns |
| CF | PN | 2023 | HybA | HybB | 2.44 | 3.29 | -0.86 | -9.74 | 1.73 | 0.02 | * |
| CF | PN | 2024 | HybA | HybB | 4.46 | 4.19 | 0.27 | 1.86 | 1.36 | 0.26 | ns |
| CF | CC | 2023 | HybA | HybB | 2.72 | 3.21 | -0.49 | -2.12 | 1.02 | 0.28 | ns |
| CF | CC | 2024 | HybA | HybB | 4.39 | 4.31 | 0.08 | 0.19 | 1.00 | 0.88 | ns |
| CF | NTPN | 2023 | HybA | HybB | 2.82 | 3.28 | -0.46 | -2.24 | 1.96 | 0.16 | ns |
| CF | NTPN | 2024 | HybA | HybB | 4.29 | 4.51 | -0.21 | -1.82 | 1.09 | 0.31 | ns |
| CF | NTCC | 2023 | HybA | HybB | 2.80 | 3.09 | -0.29 | -0.69 | 1.19 | 0.60 | ns |
| CF | NTCC | 2024 | HybA | HybB | 4.20 | 4.43 | -0.23 | -2.14 | 1.55 | 0.20 | ns |
| CF | CCPN | 2023 | HybA | HybB | 2.74 | 3.26 | -0.53 | -2.82 | 1.95 | 0.11 | ns |
| CF | CCPN | 2024 | HybA | HybB | 3.95 | 4.36 | -0.41 | -1.17 | 2.00 | 0.36 | ns |
| CF | NTCCPN | 2023 | HybA | HybB | 2.56 | 3.22 | -0.66 | -4.85 | 1.47 | 0.07 | ns |
| CF | NTCCPN | 2024 | HybA | HybB | 4.01 | 4.47 | -0.46 | -1.47 | 1.88 | 0.29 | ns |


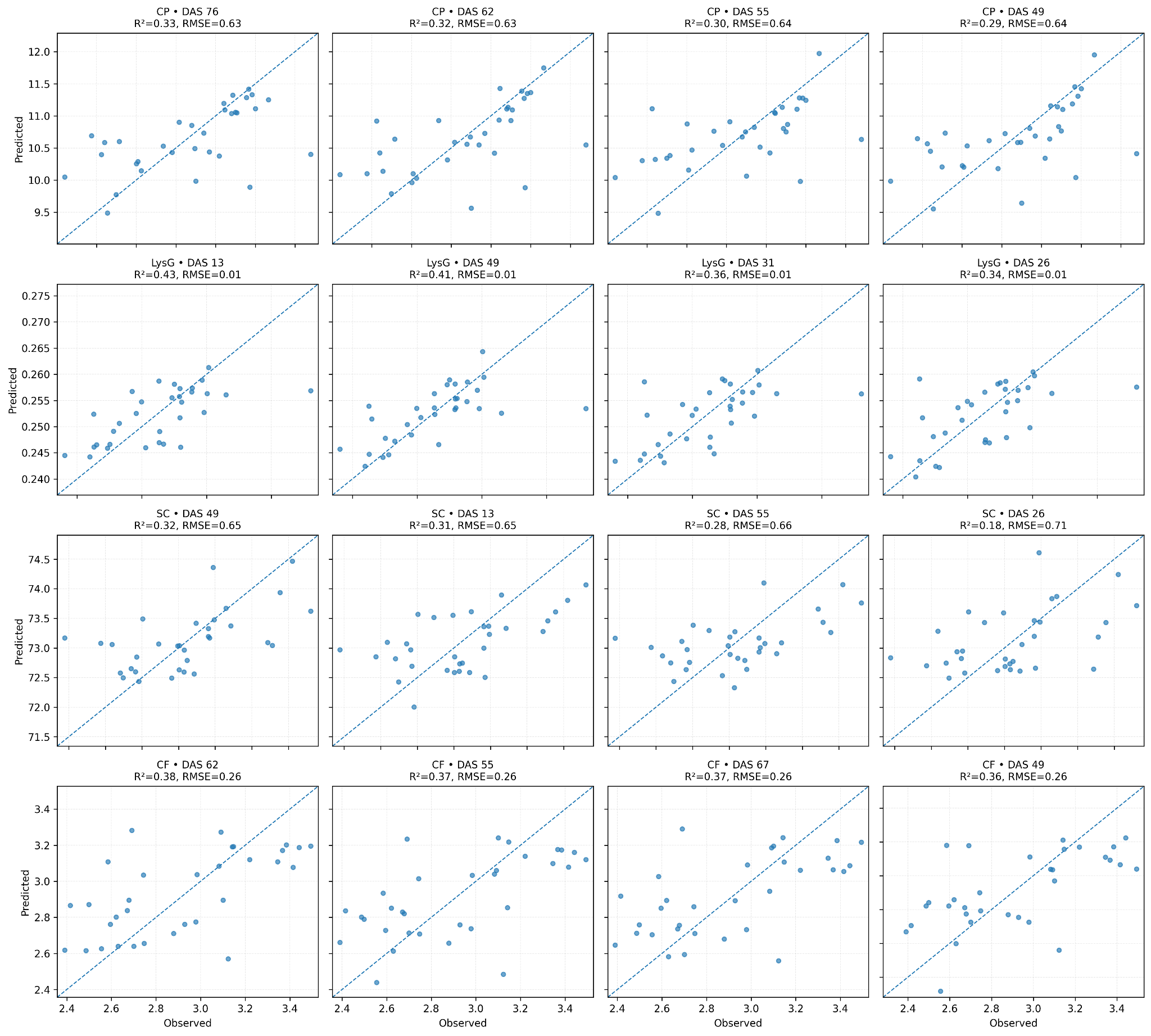


Figure S2. **Potential of UAV-Derived RGB and Multispectral Indices as Early Predictors of Key Biochemical Traits**

Each subplot shows observed vs. predicted values (Ensemble predictions) for top-performing DAS timepoints across four biochemical traits (CP, LysG, SC, CF). UAV-derived indices from RGB and multispectral (MS) sensors were evaluated as early predictors using multiple regression models.

**
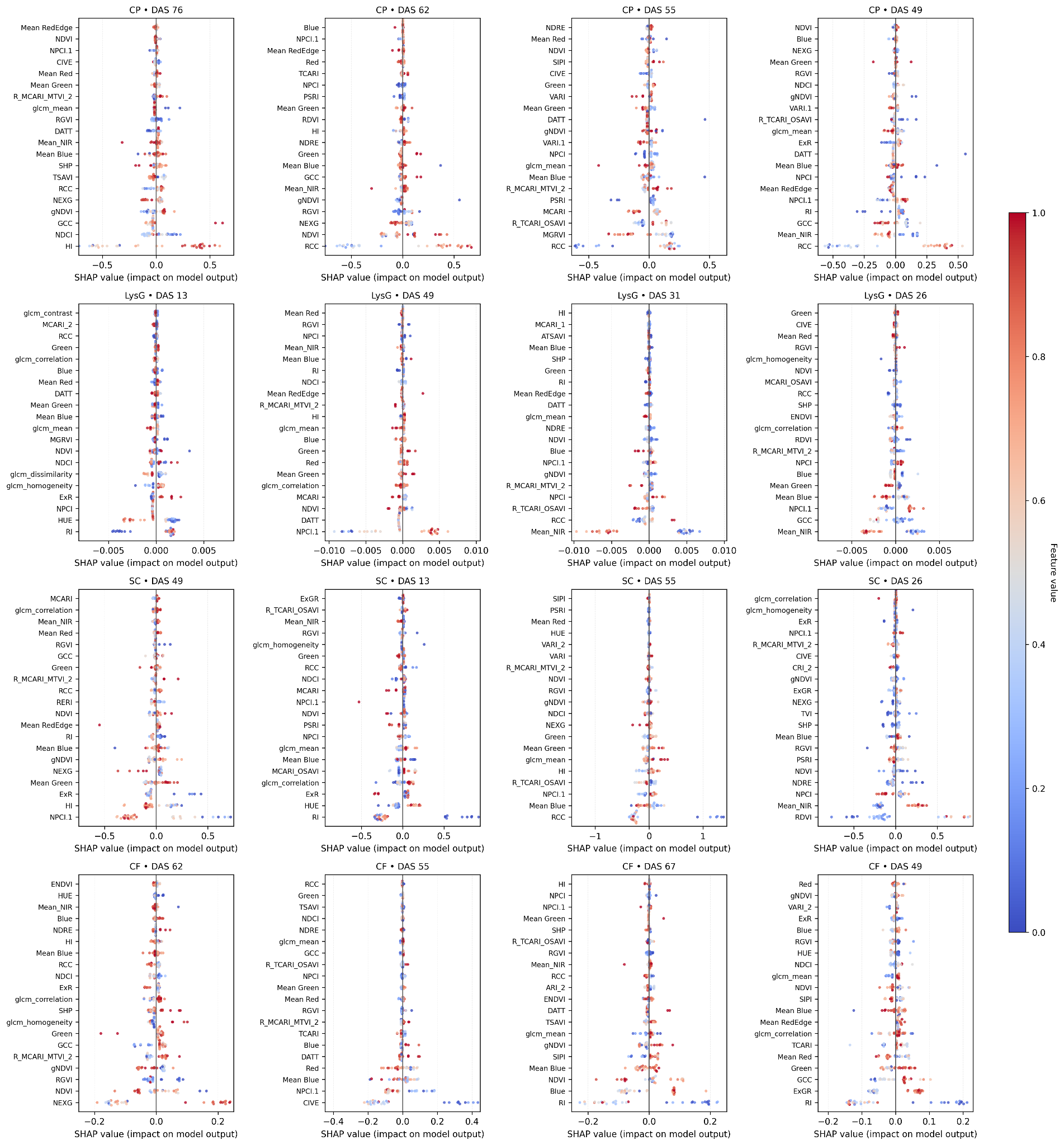
**

Figure S3. **SHAP Visualization of UAV-Derived RGB/MS Indices for Early Trait Modeling**


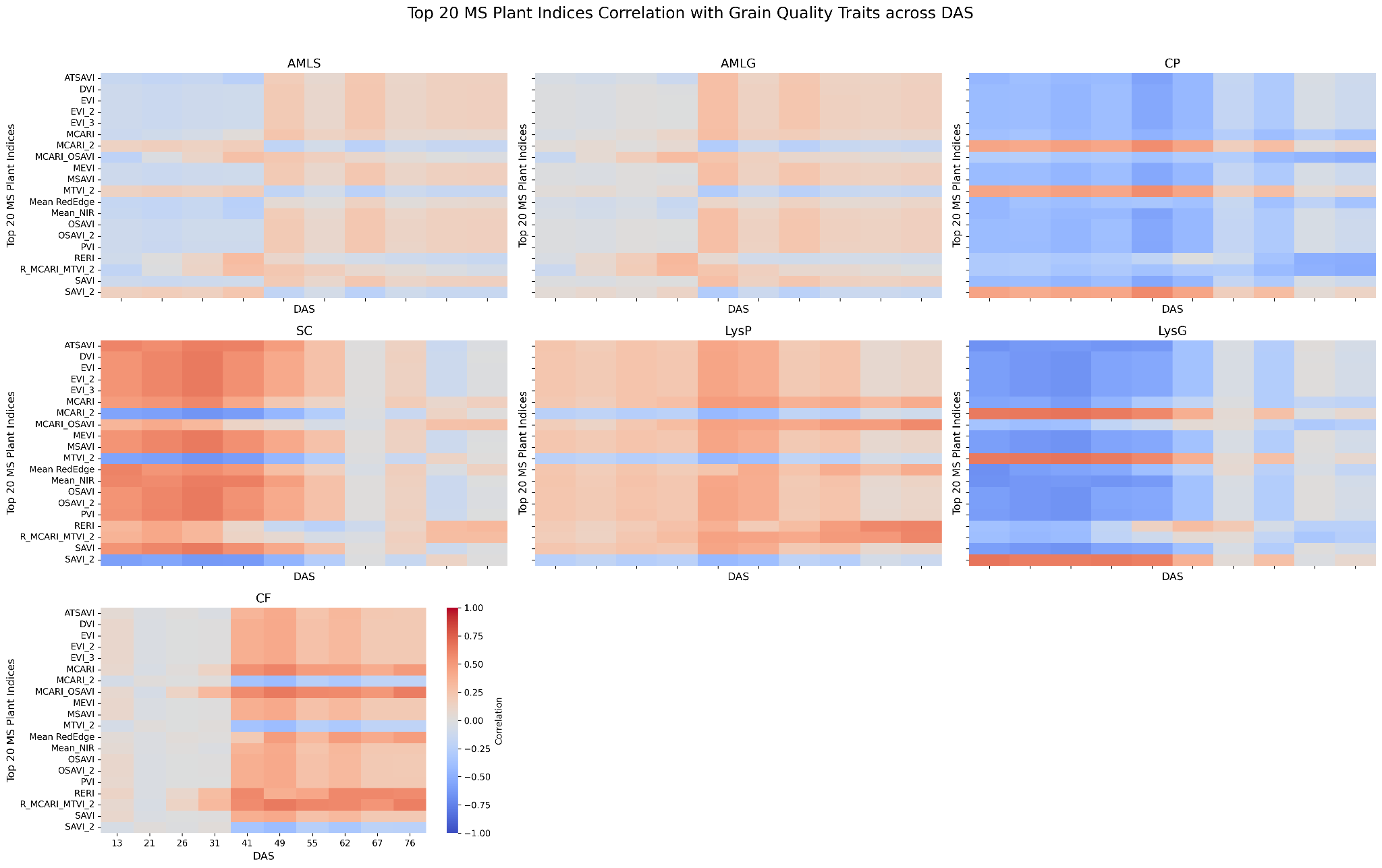


Figure S4. **Time Series Multispectral Derived Indices Correlation with Grain Quality Traits**


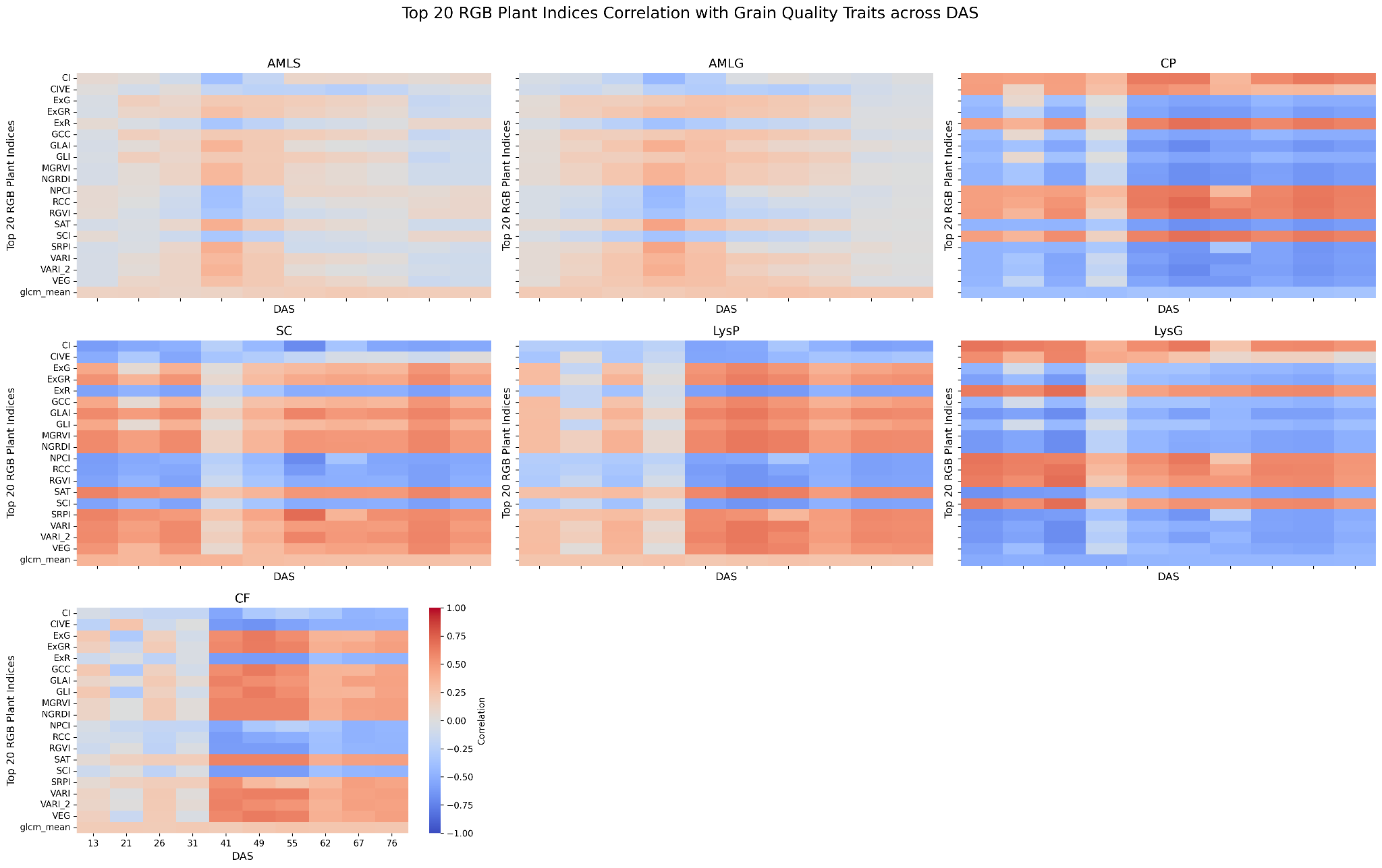


Figure S5. **Time Series RGB Derived Indices Correlation with Grain Quality Traits**

**Figure S6. Potential of Preharvest traits as Early Predictors of Key Biochemical Traits.**

Models were trained using only preharvest inputs (UAV indices, physiology, and agronomic features measured before harvest; excluding yield and grain chemistry traits) and evaluated using 5-fold cross-validation. The results show moderate predictability for CP (R^2^ = 0.49) and LysP (R^2^ = 0.67) but weak performance for amylose (R^2^ = 0.05), starch (R^2^ = 0.18), and LysG (R^2^ = 0.24), indicating that grain biochemical composition is only partially captured by early-season phenotypes. The relatively higher R² for CF (0.85) suggests stronger linkage to canopy/physiological status in this dataset but this finding should be interpreted cautiously given sample size and environmental dependence.

**-Crude Protein, Amylose from Starch, and Grain**

**
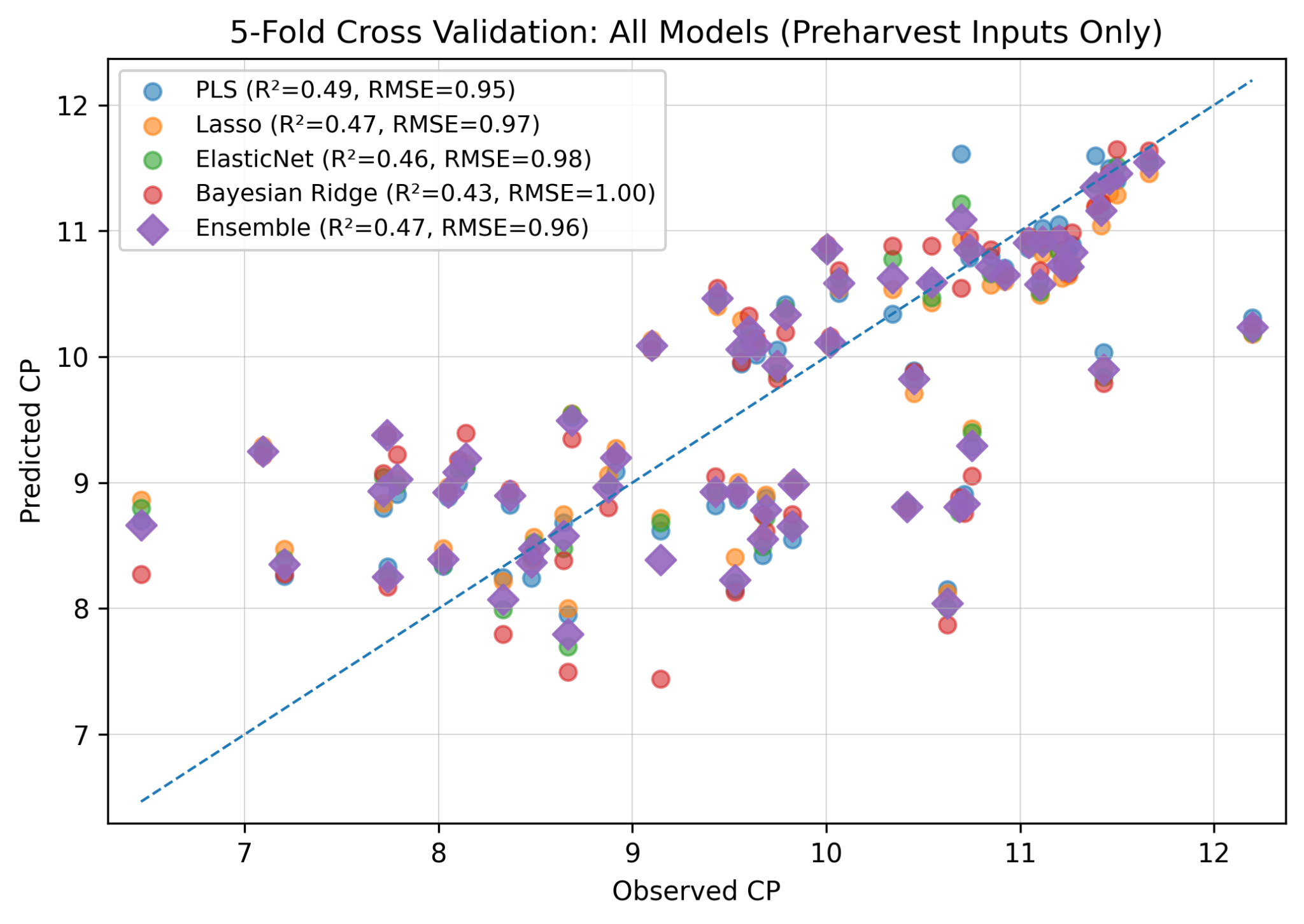

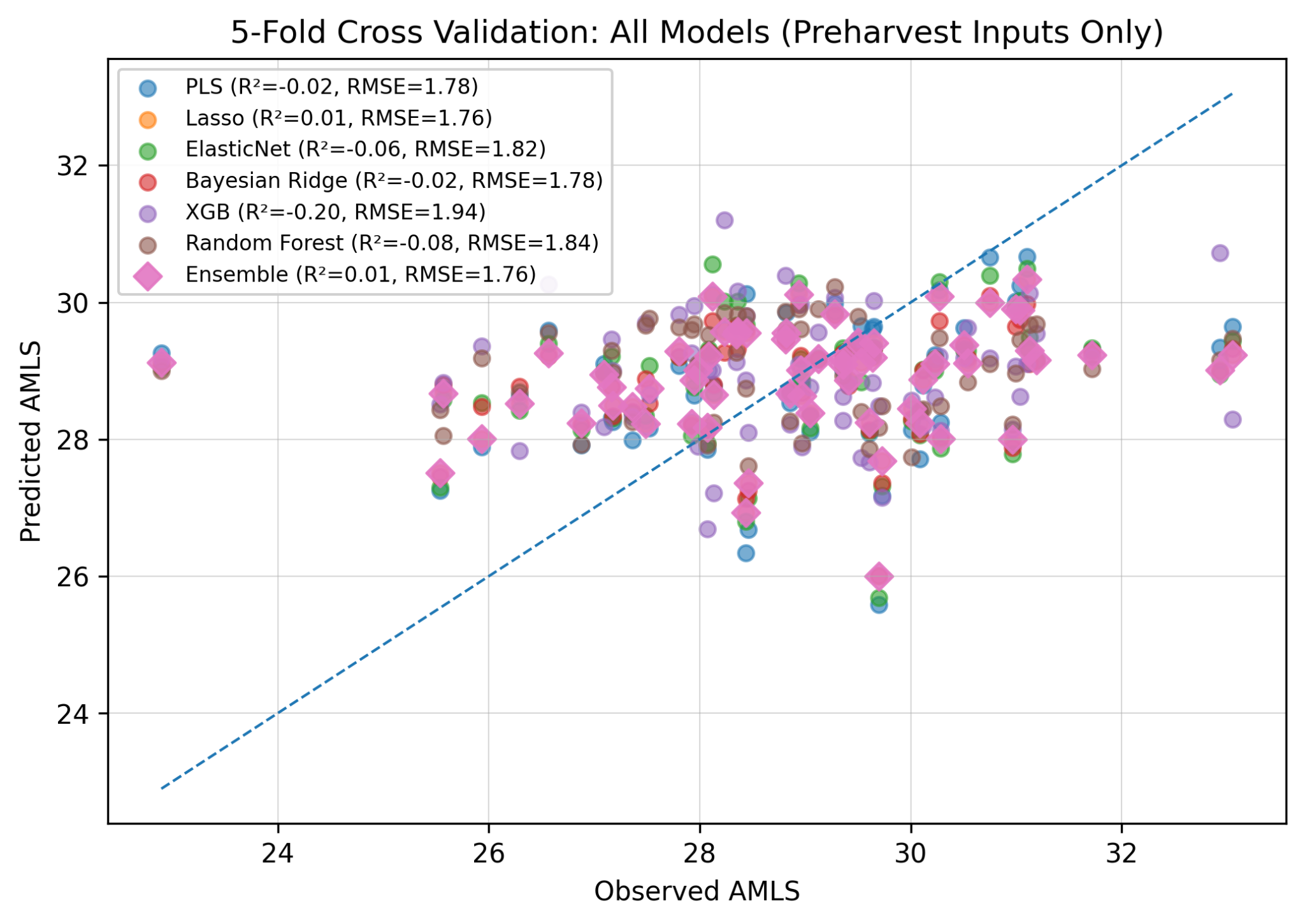

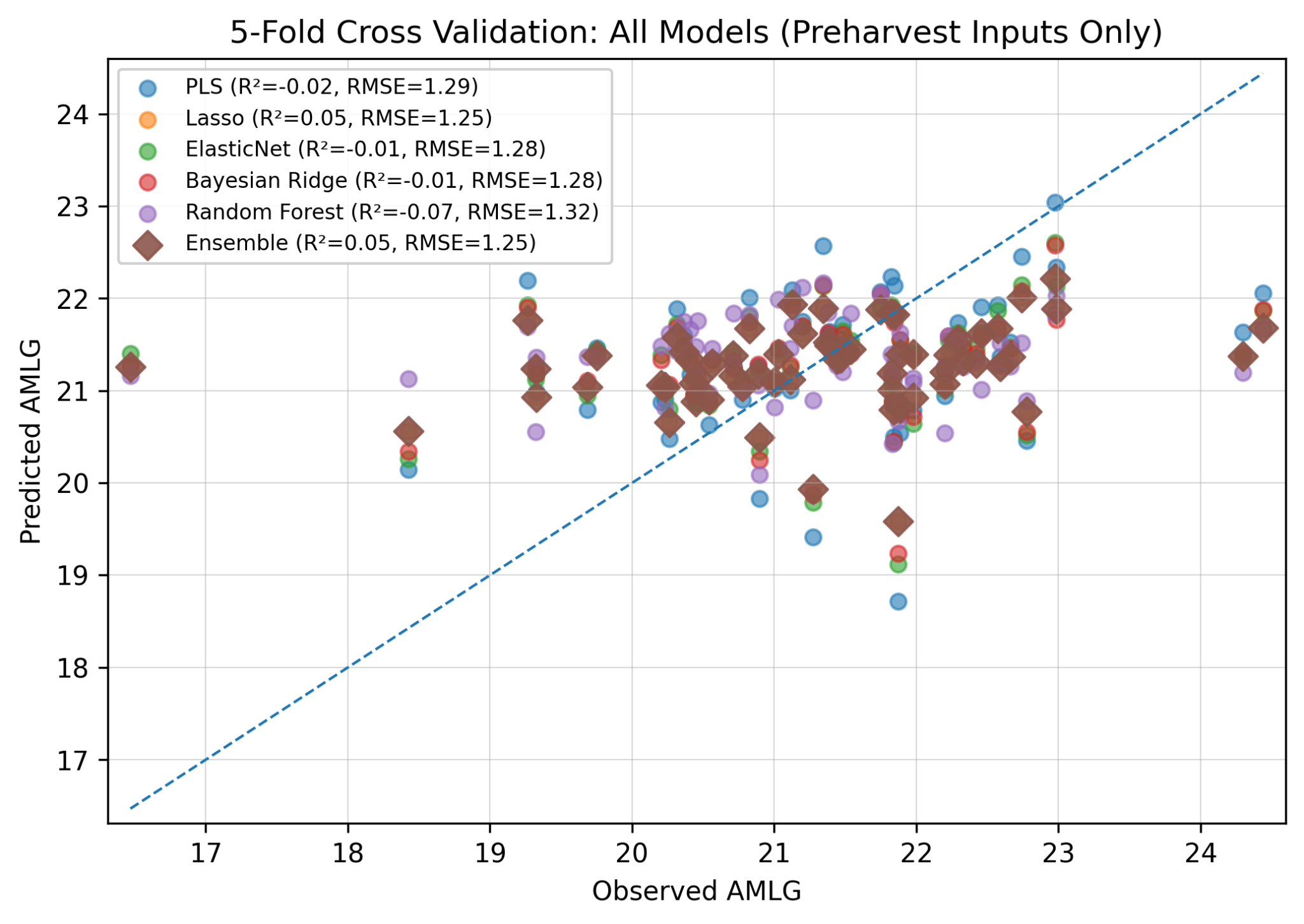
**

**-Lysine from Protein (LysP) and Starch (SC)**

**
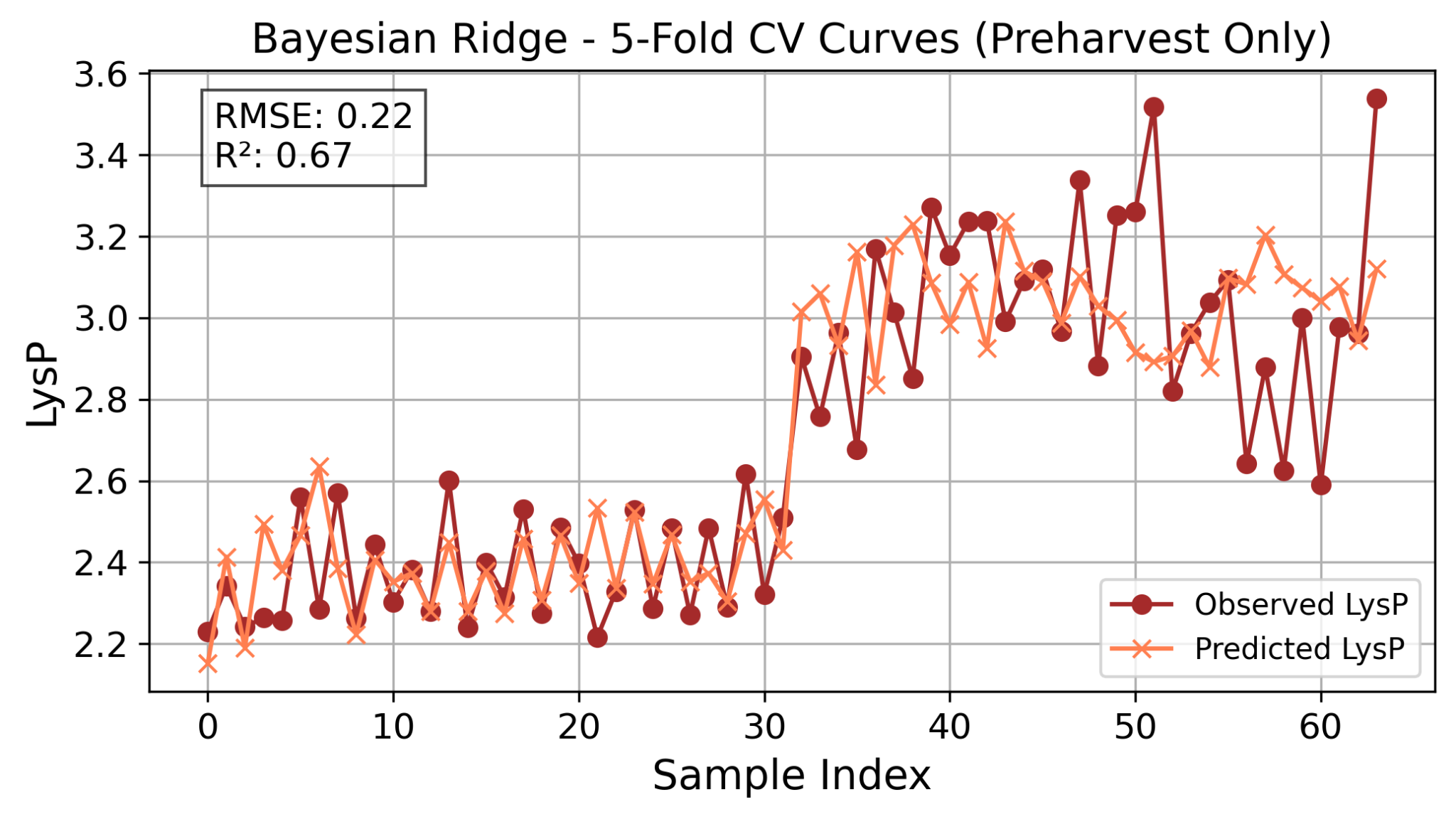

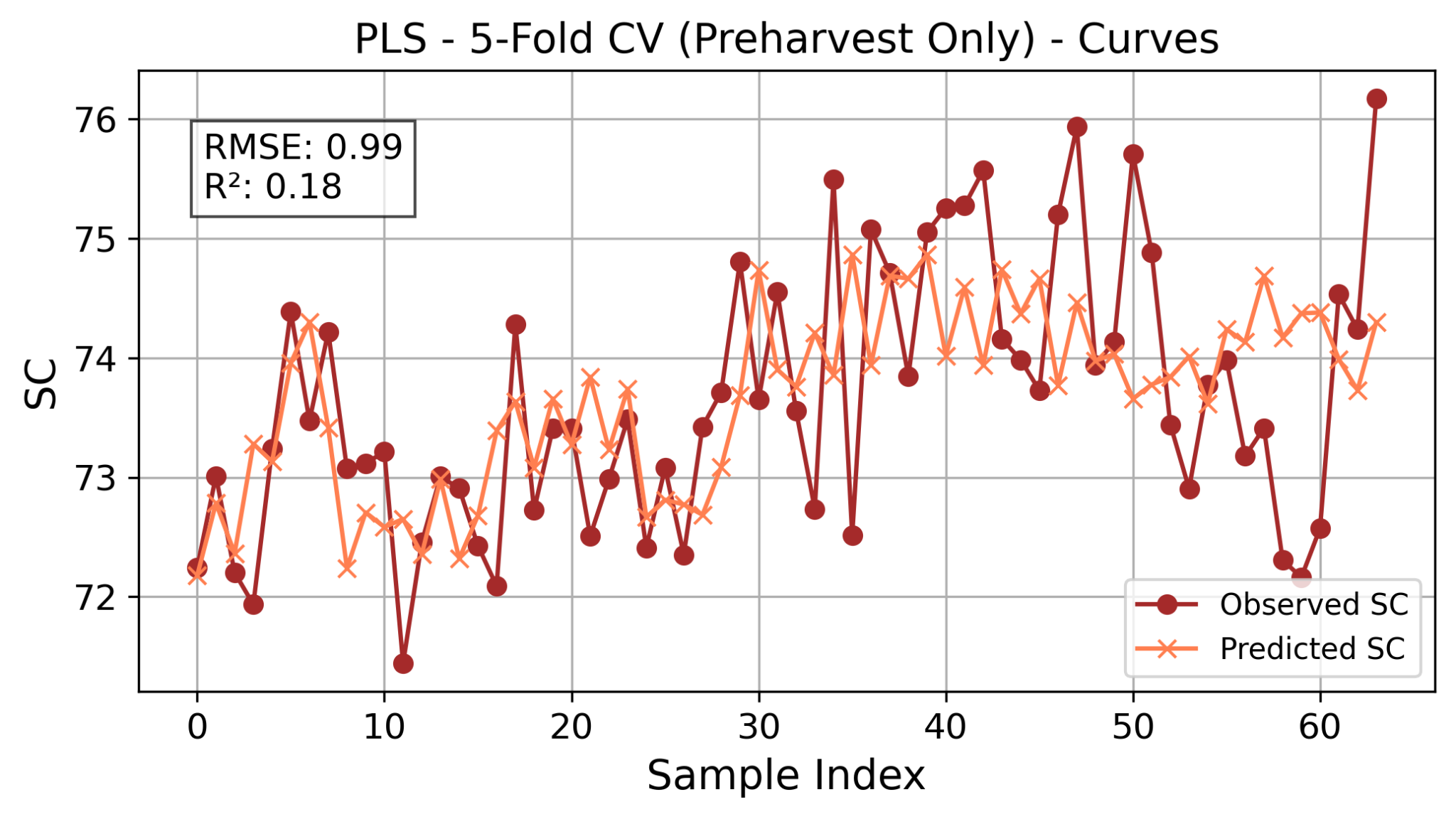
**

**-Lysine from Grain (LysG), and Crude Fat (CF)**

**
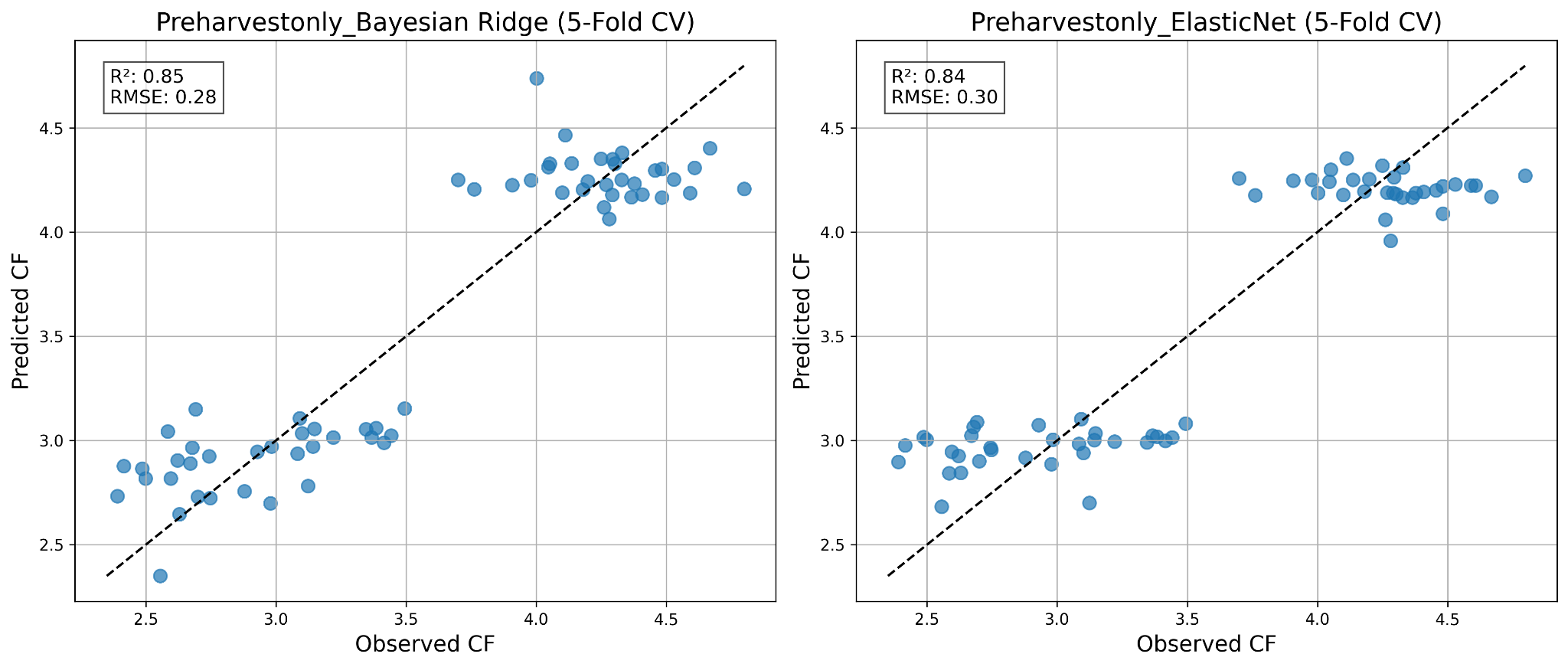

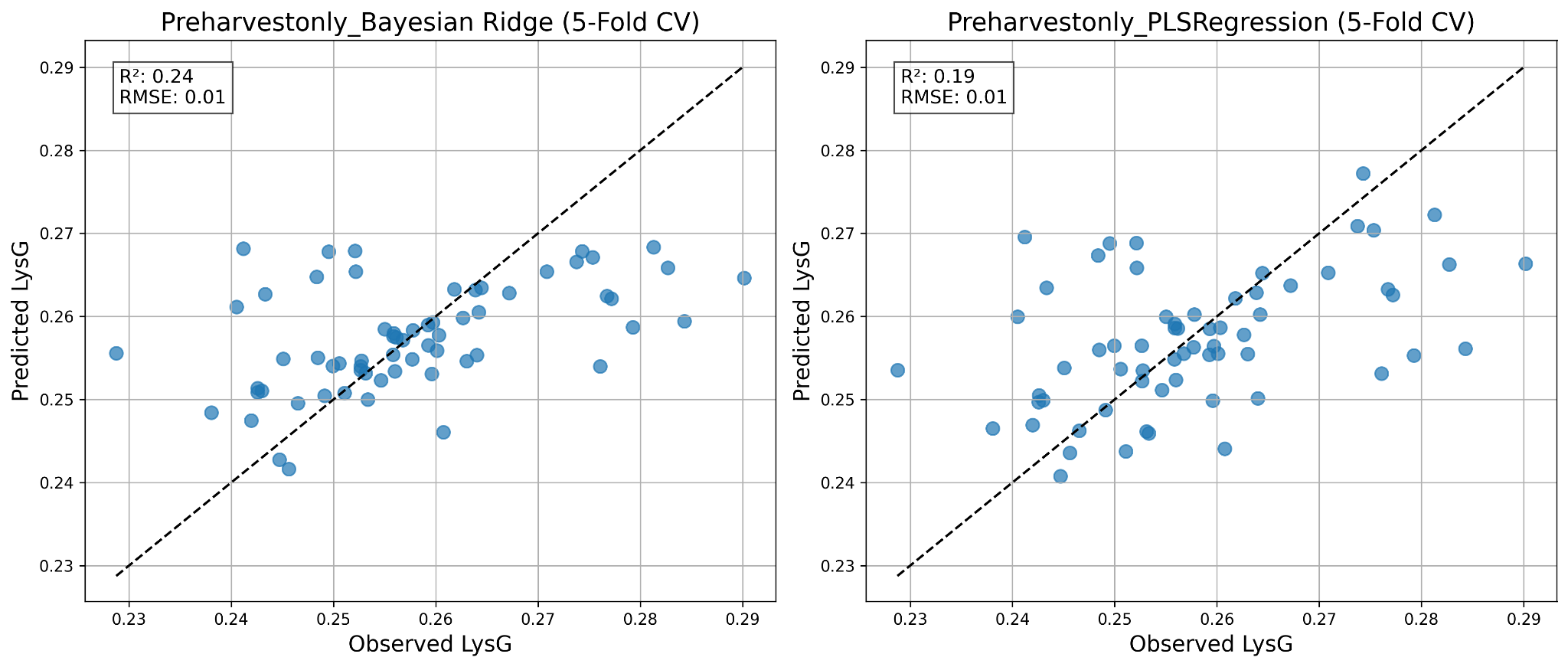
**

**Table S7. Reduction in Modelling Accuracy (R²) When Using Pre-harvest-only Features Compared to Full Feature Sets.**

| **Targets** | **all traits** | **Pre-harvest only** | **Difference** | **Decrease (%)** |
| --- | --- | --- | --- | --- |
| CP | 0.97 | 0.49 | 0.48 | 49.48 |
| AMLS | 0.99 | 0.05 | 0.94 | 94.95 |
| AMLG | 0.98 | 0.05 | 0.93 | 94.90 |
| LysG | 0.98 | 0.24 | 0.74 | 75.51 |
| LysP | 0.99 | 0.67 | 0.32 | 32.32 |
| SC | 0.82 | 0.18 | 0.64 | 78.05 |
| CF | 0.91 | 0.85 | 0.06 | 6.59 |

Supplemental Figures. Additional plots including models features shap values for temporal validation (TV), cross validation (CV), and spatial validation (SV) are available [here](https://figshare.com/s/0067a808a36370cdaf6b)

**References:**

Alphabetical List of Spectral Indices (n.d.). Available at: https://www.nv5geospatialsoftware.com/docs/AlphabeticalListSpectralIndices.html (Accessed November 5, 2025).

De Swaef, T., Maes, W. H., Aper, J., Baert, J., Cougnon, M., Reheul, D., et al. (2021). Applying RGB- and thermal-based vegetation indices from UAVs for high-throughput field phenotyping of drought tolerance in forage grasses. *Remote Sens. (Basel)* 13, 147. doi: 10.3390/rs13010147

Gitelson, A. A., Gritz, Y., and Merzlyak, M. N. (2003). Relationships between leaf chlorophyll content and spectral reflectance and algorithms for non-destructive chlorophyll assessment in higher plant leaves. *J. Plant Physiol.* 160, 271–282. doi: 10.1078/0176-1617-00887

Grbović, Ž., Ivošević, B., Budjen, M., Waqar, R., Pajević, N., Ljubičić, N., et al. (2025). Integrating UAV multispectral imaging and proximal sensing for high-precision cereal crop monitoring. *PLoS One* 20, e0322712. doi: 10.1371/journal.pone.0322712

Kataoka, T., Kaneko, T., Okamoto, H., and Hata, S. (2004). Crop growth estimation system using machine vision., in *Proceedings 2003 IEEE/ASME International Conference on Advanced Intelligent Mechatronics (AIM 2003)*, (IEEE). doi: 10.1109/aim.2003.1225492

List of available Indices (n.d.). Available at: https://www.indexdatabase.de/db/i.php (Accessed November 5, 2025).

Louhaichi, M., Borman, M. M., and Johnson, D. E. (2001). Spatially located platform and aerial photography for documentation of grazing impacts on wheat. *Geocarto Int.* 16, 65–70. doi: 10.1080/10106040108542184

Memon, M. S., Chen, S., Guo, J., Iqbal, B., Du, Z., Taha, M. F., et al. (2025). Estimating rice yield under different wheat residue coverage levels using multispectral Gaofen satellite data and remote sensing indices. *J. Agric. Eng.* 56. doi: 10.4081/jae.2025.1698

Radócz, L., Szabó, A., Tamás, A., Illés, Á., Bojtor, C., Ragán, P., et al. (2023). Investigation of the detectability of corn smut fungus (Ustilago maydis DC. Corda) infection based on UAV multispectral technology. *Agronomy (Basel)* 13, 1499. doi: 10.3390/agronomy13061499

Reudenbach, C. (2019). rgbi: RGB indices in gisma/robubu: the rolling burning bus - whatever, it runs fine for now. Available at: https://rdrr.io/github/gisma/robubu/man/rgbi.html (Accessed March 17, 2025).

rgb_indices function - RDocumentation (n.d.). Available at: https://www.rdocumentation.org/packages/uavRst/versions/0.5-4/topics/rgb_indices (Accessed March 17, 2025).

Shammi, S. A., Huang, Y., Feng, G., Tewolde, H., Zhang, X., Jenkins, J., et al. (2024). Application of UAV multispectral imaging to monitor soybean growth with yield prediction through machine learning. *Agronomy (Basel)* 14, 672. doi: 10.3390/agronomy14040672
